# Synergistic Mechano‐Fluidic Self‐Fusion of Poly(Vinyl Alcohol)‐Modified Liquid‐Metal Particles for Post‐Treatment‐Free Soft Electronics

**DOI:** 10.1002/advs.78062

**Published:** 2026-10-06

**Authors:** Keun‐Young Yook, Sangwon Kim, Chansoo Kim, Minkyong Kang, Yurim Lee, Mido Kim, Kijun Park, Seung Yeop Han, Seung‐Woo Cho, Gun‐Hee Lee, Kayoung Son, Hanbit Jang, Tae Young Kim, Jungmok Seo

**Affiliations:** ^1^ School of Electrical and Electronic Engineering Yonsei University Seoul Republic of Korea; ^2^ Department of Medicine Brigham and Women's Hospital Harvard Medical School Massachusetts USA; ^3^ Department of Biotechnology Yonsei University Seoul Republic of Korea; ^4^ Department of Biomaterials Science and Engineering Yonsei University Seoul Republic of Korea; ^5^ CellArtgen Inc. Seoul Republic of Korea; ^6^ Center for Nanomedicine Institute for Basic Science (IBS) Seoul Republic of Korea; ^7^ Department of Biomedical Engineering Ulsan National Institute of Science and Technology (UNIST), UNIST‐gil 50 Ulju‐gun Ulsan Republic of Korea

**Keywords:** enhanced wettability, instant conductivity, Marangoni‐driven self‐fusion, polymer‐shelled LMP, post‐treatment‐free

## Abstract

Liquid metals (LMs) are promising candidates for soft and stretchable conductors; however, their high surface tension and poor wettability pose significant challenges for high‐resolution printing. To address these limitations, LMs are commonly processed into liquid metal particles (LMPs), which improve printability. Nevertheless, the insulating oxide shell surrounding each particle persists, typically necessitating additional post‐treatment steps to achieve electrical conductivity. Here, we report synergistic mechano‐fluidic self‐fusion of poly(vinyl alcohol) (PVA)‐modified liquid‐metal particles (PmLMPs) for post‐treatment‐free soft interconnects. A sprayable PmLMP ink is formulated in which an ultrathin PVA shell stabilizes the particles and enhances substrate adhesion through hydrogen bonding. Direct electrical interconnection is achieved via a two‐stage process: spray‐induced mechanical impact ruptures the oxide shells to initiate metallic contact, followed by binary‐solvent‐driven Marangoni convection (deionized (DI) water/ethanol = 3:1 v/v) that promotes particle rearrangement and metallic neck growth. This mechano‐fluidic sequence enables rapid formation of conductive networks without post‐treatment. The printed films exhibit an initial conductivity of 1.5 × 10^6^ S m^−^^1^, maintain electrical continuity under monotonic strains exceeding 200% and during approximately 1,400 stretch‐release cycles at 100% strain, and support 50 µm‐scale patterning with large‐area scalability. This work establishes a flow‐assisted metallic fusion approach for scalable, instantly conductive soft interconnects.

## Graphical abstract

Poly(vinyl alcohol)‐modified liquid‐metal particles form conductive networks through spray‐induced self‐fusion without additional post‐treatment. Spray impact initiates oxide‐shell rupture and electrical contact, while subsequent Marangoni flow and capillary forces promote particle redistribution, neck growth, and network consolidation. This sequential mechanism enables direct printing of conductive interconnects in a single deposition step for soft electronics.

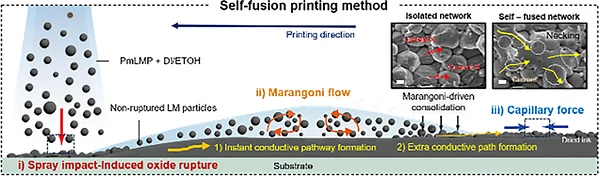

## 1 Introduction

Wearable and soft electronic technologies have rapidly evolved as essential platforms for healthcare monitoring, electronic skin, and human–machine interfaces [1, 2, 3, 4, 5, 6]. Their success, however, hinges on the development of electronic materials that can intimately conform to soft biological tissues while maintaining reliable electrical performance over repeated mechanical deformation [7, 8]. Human skin and organs typically exhibit Young's moduli in the kilopascal (kPa) range, in contrast to the gigapascal (GPa) stiffness of conventional electronic materials [9, 10]. This pronounced mechanical mismatch causes delamination, cracking, and eventual electrical failure during stretching or bending, limiting the durability and comfort of long‐term wearable devices [11, 12, 13]. Therefore, developing soft interconnects combining metallic‐level conductivity with mechanical compliance and large‐area manufacturability is a fundamental challenge for realizing truly skin‐conformal electronics.

Traditional metallic materials, such as copper or aluminum, offer excellent electrical conductivity but undergo fracture or irreversible damage under large mechanical deformation [14, 15, 16, 17]. Geometric designs, such as serpentine or fractal layouts, can impart stretchability to rigid metals but require complex fabrication and limit conductor density and routing freedom. Flexible nanomaterial‐based conductors offer an alternative route with improved mechanical compliance and transparency, but they often sacrifice metallic‐level conductivity. These trade‐offs highlight the need for an intrinsically deformable conductor that can sustain high electrical conductivity under strain and be processed through scalable, low‐cost manufacturing routes compatible with diverse substrates. Gallium‐based liquid metals (LMs) have emerged as promising intrinsically soft and stretchable conductors because they combine metallic conductivity (∼10^6^ S m^−^^1^) with liquid‐like deformability at room temperature, allowing them to endure extreme mechanical strain without cracking [18, 19, 20, 21, 22]. However, the widespread adoption of LMs in practical soft interconnects remains limited by several inherent challenges. Upon exposure to air, gallium readily forms a self‐limiting oxide layer (∼3 nm thick) that stabilizes the droplet morphology but acts as an electrical insulator [23, 24]. This oxide layer inhibits electron transport and coalescence between neighboring particles. Moreover, their very high surface tension (∼600 mN m^−^^1^) impedes wetting and uniform patterning on elastomeric substrates [18, 25]. Thus, most LM‐based inks remain electrically non‐conductive after deposition and require an additional activation or treatment step (e.g., thermal, mechanical, or laser treatment) to rupture the oxide shell and establish conductive pathways [18, 19, 26].

Liquid metal particles (LMPs) generated by ultrasonication or shear mixing exhibit reduced effective surface tension and improved printability [19, 20, 27, 28, 29]. The incorporation of surface modifiers such as catechol‐rich tannic acid (TA) and poly(vinyl alcohol) (PVA) has been widely investigated to improve dispersion stability, adhesion, and process compatibility [30, 31]. Prior PVA–LM systems used PVA to improve wettability and 3D printability in an ink requiring mechanical turn‐on, to create a borate‐crosslinked magnetically responsive self‐healing GaInSn–Ni hydrogel, or to form a tough laminated PVA hydrogel with a sedimentation‐derived LM‐rich conductive layer; however, none combined spray‐impact rupture of oxide/PVA‐shelled particles with evaporation‐driven consolidation in a single post‐treatment‐free deposition step, as pursued here [32, 33, 34]. Evaporation‐induced and self‐sintering strategies have also been developed to form conductive LM networks during drying [35, 36, 37, 38, 39, 40]. Direct spray approaches have also been reported: Wang and Liu atomized already‐conductive bulk GaIn through a mask, whereas Zhang et al. used ultrasonic atomization to form LM microparticles for deformable contacts in an elastomeric circuit platform [41, 42]. Unlike these bulk‐ or atomization‐based approaches, the present system begins with initially nonconductive oxide/PVA‐shelled particles and couples impact‐gated shell rupture with binary‐solvent‐assisted consolidation during deposition.

Here, we introduce PVA‐modified LM particles (PmLMPs) via synergistic mechano‐fluidic self‐fusion, enabling post‐treatment‐free, deposition‐induced sintering of LMPs during spray deposition. This mechanism integrates three synergistic processes into a continuous sequence. First, high‐velocity impact of aerosolized particles produces sufficient mechanical stress to rupture the thin gallium (III) oxide (Ga_2_O_3_) shell, exposing fresh metallic surfaces and allowing immediate particle‐particle contact [43]. Second, each particle is coated with an ultrathin PVA shell (7–10 nm), which prevents rapid reoxidation and mediates adhesion and bridging between adjacent LMPs. Because the PVA layer is extremely thin, it stabilizes the interface between LMPs without hindering electron transport, functioning as a fusion mediator rather than an insulating barrier. Third, evaporation of an ethanol–water binary solvent induces a surface‐tension gradient that drives Marangoni convection, redistributing particles, promoting additional collisions, and facilitating metallic neck growth through capillary consolidation [44, 45]. By coupling these processes within a single spray‐deposition event, conductive networks form without separate thermal, mechanical, laser or chemical activation. The resulting films exhibit uniform morphology, strong interfacial adhesion, and immediate electrical percolation, establishing a new pathway for scalable, post‐treatment‐free fabrication of soft conductors. Rather than claiming a universal solution to LMP sintering, this work provides a practical materials‐processing strategy for forming conductive, mechanically compliant LM networks by direct spray patterning.

The self‐fusion process produces metallic pathways that retain both high conductivity and mechanical resilience under strain. Because the polymer shell is ultrathin and chemically benign, it also offers biocompatibility and stable adhesion to diverse substrates, including elastomers, plastics, and hydrogels [46, 47]. The films fabricated through this process exhibit an electrical conductivity of approximately 1.5 × 10^6^ S m^−^^1^ immediately after spraying, maintaining electrical continuity under monotonic strains exceeding 200% and during approximately 1,400 stretch‐release cycles at 100% strain. The spray deposition method enables precise patterning with nominal linewidth down to 50 µm and uniform coating over areas larger than 20 × 20 cm^2^, demonstrating its scalability and batch‐compatible manufacturing potential. The sprayed traces reliably powered light‐emitting diodes (LEDs) during repeated stretching, bending, and twisting without electrical failure. To further demonstrate scalability, we fabricated large‐area patterns directly onto textile surfaces and integrated them as surface electromyography (EMG) sensors, enabling real‐time control of a remote‐controlled vehicle through muscle‐activation signals. These results highlight the robustness, deformability, and practical applicability of our PmLMP‐based spray‐printing approach for soft and wearable electronic systems.

## 2 Results

### 2.1 Self‐Fusion Spray Deposition of PmLMPs for Scalable Soft‐Electronic Fabrication

Sonication of bulk Eutectic Gallium‐Indium (EGaIn) produces LMPs that are immediately encapsulated by Ga_2_O_3_ oxide shell [48]. Although this oxide layer provides only modest improvement in wettability compared to the bulk LM, the resulting particles still fail to spread adequately on soft substrates and readily aggregate. To overcome this limitation, we fabricated PmLMPs by introducing PVA during sonication (Figure 1a). The PVA chains interact with Ga_2_O_3_ oxide shell of LMPs through hydrogen bonding, forming a PVA‐mediated interfacial layer that lowers the particle surface energy (Figure 1b). This modification enables the PmLMPs to spread more uniformly on soft substrates with strong adhesion (Figure 1c) and resist strain‐induced aggregation, maintaining stable particle arrangements even under deformation. Beyond improved wettability, the PVA also facilitates particle–particle fusion. As adjacent PmLMPs come into contact, metallic coalescence occurs in parallel with polymer bridging between their oxide shells, promoting neck formation and stabilizing a continuous conductive network (Figure 1d).

**FIGURE 1:**
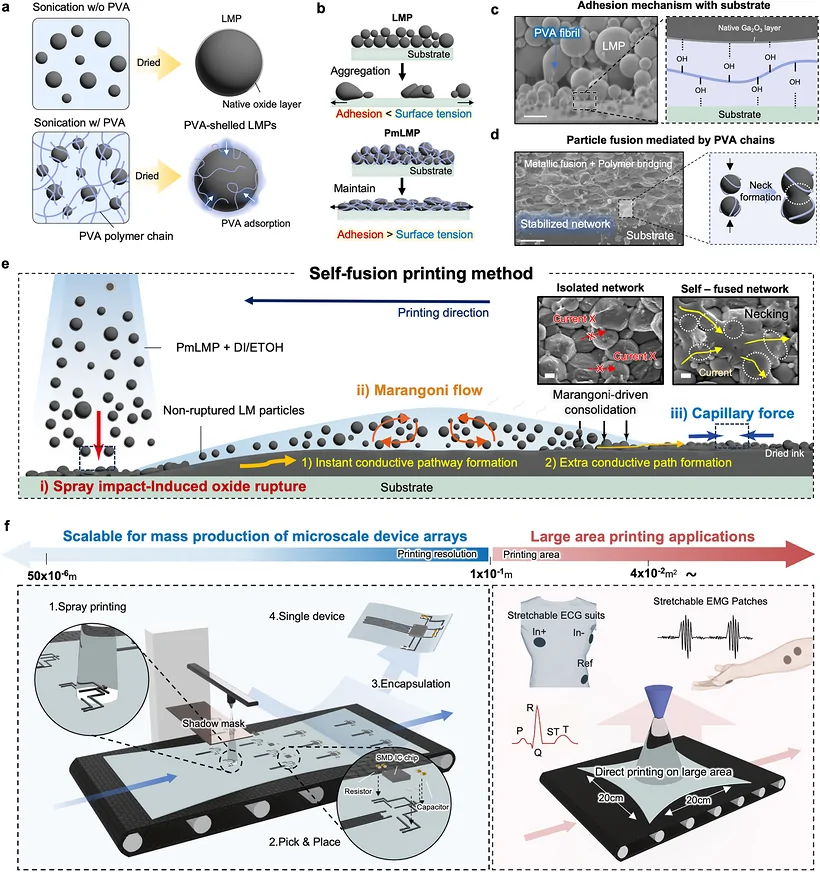
PmLMP for soft electronics. a Formation of LMP by sonicating bulk EGaIn with and without PVA. b Wetting behavior of LMP and PmLMP on soft substrates. c Representative SEM image and schematic illustration of the PVA‐mediated interfacial layer. (Scale bar = 2 µm) d Representative SEM image and schematic illustration of particle fusion and network stabilization, where metallic necking and polymer bridging contribute to conductive‐network formation. (Scale bar = 5 µm) e Schematic overview of the self‐fusion spray‐printing process. Conductive‐network formation proceeds through sequential spray‐impact activation, hydrodynamic redistribution/ consolidation, and capillary‐assisted necking during drying. The inset SEM images compare an isolated particle network with a self‐fused particle network formed after spray printing. (Scale bars = 1 µm) f Schematic overview of the scalability of the process, spanning microscale device‐array fabrication and large‐area wearable/bioelectronic applications.

Based on this optimized particle interface, we developed a self‐fusion spray‐printing process in which conductive pathways are formed without additional post‐treatment (Figure 1e). In this process, conductive‐network formation is proposed to occur through three cooperative steps: spray‐impact‐induced activation, hydrodynamic redistribution/ consolidation, and capillary‐assisted necking during drying. The spray impact first promotes local rupture of the oxide/PVA shell and initiates electrical percolation. Subsequent solvent‐driven flow and capillary contraction further promote particle redistribution, interparticle contact, and metallic neck formation, resulting in a continuous conductive network. These combined effects enable PmLMP to self‐fuse spontaneously during the spray process, producing conductive traces without the need for any external sintering step. The post‐treatment‐free nature of the PmLMP spray process enables straightforward integration into scalable manufacturing workflows (Figure 1f). Using a simple shadow‐mask–assisted spray step, microscale circuit arrays can be continuously patterned. After printing, standard electronic components such as resistors, capacitors, and integrated circuits can be pick‐and‐placed directly onto the traces without any additional activation. This allows rapid fabrication of fully functional soft‐electronic devices with minimal processing requirements. Additionally, the PmLMP ink can be directly printed over textile surfaces to create wearable electrocardiography (ECG) or EMG interfaces that operate immediately after printing. These demonstrations illustrate that the proposed strategy can bridge microscale device‐array fabrication and large‐area wearable systems, providing a practical route toward scalable soft‐electronic applications.

### 2.2 Fabrication Process of PmLMP and its Characterization

Figure 2 presents the pre‐spray optimization and characterization of the PVA‐modified liquid metal particle ink, including particle fabrication, shell‐structure control, interparticle contact, adhesion, and deformation‐related morphology. Figure 2a illustrates the fabrication workflow and particle‐level evolution of PmLMP prior to spray deposition, from liquid metal fragmentation to polymer‐shell optimization for self‐fusion‐enabled printing. To generate PVA‐shelled liquid metal particles, bulk EGaIn was ultrasonicated in a 5 wt% PVA aqueous solution. Sonication was performed at 20% amplitude for a total of 10 min using a pulsed mode (5 s on / 5 s off) to prevent overheating [49]. During ultrasonication, the bulk metal was efficiently fractured into microscale droplets, while PVA chains simultaneously interacted with the freshly formed Ga_2_O_3_ oxide shell, yielding a PVA_LMP dispersion surrounded by an excessively thick polymer layer. Particle‐level transmission electron microscopy (TEM) analysis confirmed the presence of this thick PVA coating, which substantially improved wettability but impeded direct particle‐particle contact (Figure 2a‐ii). Because close interparticle contact is essential for liquid metal self‐fusion, the excessive PVA layer suppressed conductive pathway formation, resulting in electrically insulating behavior. To obtain a particle structure suitable for self‐fusion and electrical conductivity, the PVA_LMP suspension was centrifuged at 2,000 rpm for 3 min to remove free, unbound PVA. After discarding the supernatant, the collected particles‐denoted as PmLMP‐exhibited a thinner and more uniform PVA shell (Figure 2a‐iii). This controlled ultrathin polymer layer effectively reduced surface energy and enhanced wettability while still permitting particle‐particle fusion.

**FIGURE 2:**
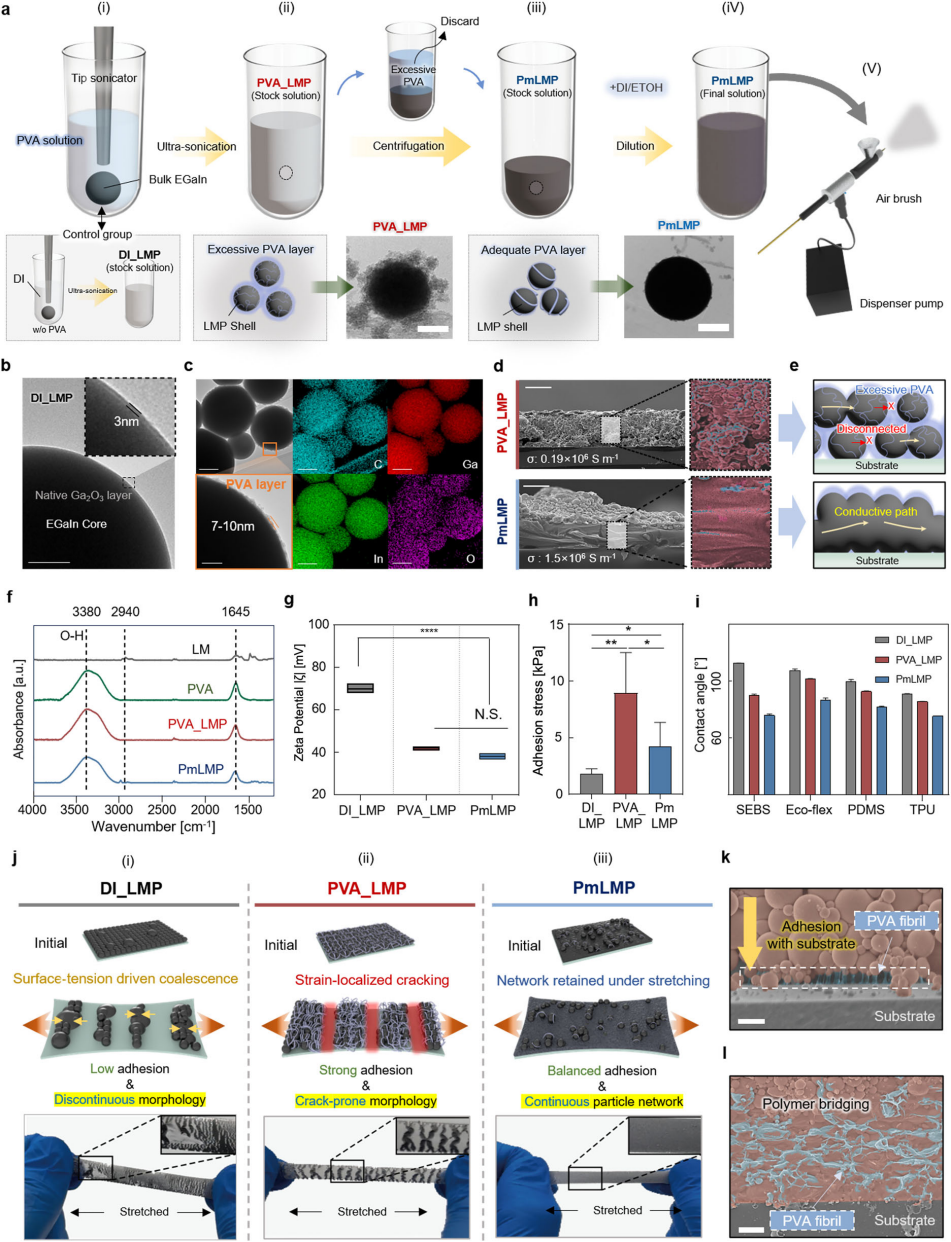
Pre‐spray optimization and characterization of PVA‐modified liquid metal particles. a Ink‐optimization workflow and enlarged TEM images comparing the morphologies of over‐coated PVA_LMP and optimized PmLMP after centrifuge refinement (scale bars = 50 nm). b TEM image of a pristine DI_LMP particle, showing a smooth ∼3 nm native oxide shell without an organic layer. (scale bars = 50 nm). c TEM image and corresponding EDS elemental mapping of PmLMP (scale bars: 200 nm for the overview TEM/EDS images and 50 nm for the magnified PVA‐layer inset). d Cross‐sectional SEM images comparing the microstructure of spray‐coated films (scale bar = 10 µm). e Schematic representation illustrating the hindrance of electron transport by excess PVA (top) vs. the formation of conductive pathways in PmLMP (bottom). f FT‐IR spectra of bulk LM, pure PVA, PVA_LMP, and PmLMP, confirming the presence of PVA functional groups on the modified particles. (n = 3) g Zeta potential measurements indicating the modulation of surface charge and electrostatic repulsion after PVA coating. (n = 3) h Adhesion stress measurements of the particles on a substrate. (n = 3) i Contact angle analysis on various stretchable substrates (styrene‐ethylene‐butylene‐styrene (SEBS), Ecoflex, polydimethylsiloxane (PDMS), and thermoplastic polyurethane (TPU)), demonstrating improved wettability of PmLMP. (n = 2) j Morphological comparison under stretching: (i) DI_LMP (low adhesion, liquid‐like beading), (ii) PVA_LMP (strong adhesion, brittle cracking), and (iii) PmLMP (balanced adhesion, stable viscoelastic deformation). k Cross‐sectional SEM image showing PVA fibrils bridging the PmLMP particles and substrate, supporting interfacial adhesion. (scale bar = 4 µm). l SEM image of the spray‐printed PmLMP network, showing PVA fibrils and polymer bridges between neighboring particles. (scale bar = 2 µm). In h and i, data are presented as mean ± SD, and error bars indicate SD (n = 3 and n = 2 per condition, respectively). Comparisons in g and h were analyzed using one‐way ANOVA followed by Tukey's multiple‐comparisons test. Adjusted p values are indicated by *p < 0.05, **p < 0.01, and ****p < 0.0001; N.S., not significant. No inferential comparison is reported for f or i.

The resulting PmLMP stock dispersion was subsequently diluted with a deionized (DI) water/ethanol (3:1 v/v) binary solvent to formulate a sprayable ink, which was directly loaded into an air‐brush system for printing. Consistent with this fabrication workflow, we present photographic and SEM evidence of the corresponding solution states prior to spraying (Figure S1a) and the morphologies of spray‐printed films obtained after air‐brush deposition (Figure S1b–c). To confirm PmLMP's structure and composition, we performed transmission electron microscopy (TEM) imaging and energy‐dispersive X‐ray spectroscopy (EDS) elemental mapping. First, we confirmed DI_LMP as control group, prepared without PVA. The TEM micrographs revealed that the DI_LMP particles possessed a smooth, spherical morphology with a distinct native oxide shell (~3 nm) but lacked any organic coating layer (Figure 2b, Figure S2) [50, 51]. In contrast, the PmLMP exhibited a different interfacial structure (Figure 2c). In the EDS maps of the PVA‐treated samples, the oxygen signal (purple) was localized around the particle surfaces, indicating the presence of the Ga_2_O_3_ oxide shell. Crucially, the carbon signal (cyan), corresponding to the PVA coating, also appeared at the particle periphery and overlapped with the oxide‐rich region. This overlap suggests that the PVA layer conformally wraps the oxide shell, consistent with a thin, well‐controlled PVA interface that maintains particle functionality. To clarify the fate of PVA in the final samples, we note that PVA is retained after deposition and solvent evaporation rather than being removed or sacrificed. The retained PVA forms an ultrathin solid interfacial layer surrounding the Ga_2_O_3_ shell, thereby contributing to particle–particle bridging and substrate adhesion while still allowing metallic percolation through the ruptured and necked liquid‐metal domains.

The 3:2 formulation ratio denotes 3.0 g EGaIn mixed with 2.0 g of a 5 wt% aqueous PVA stock, not 3.0 g EGaIn mixed with 2.0 g dry PVA. Thus, only 0.10 g PVA is introduced initially, whereas 1.90 g is DI water used as a temporary sonication medium. After centrifugation and supernatant removal, the recoverable wet mass decreased from 4.800 ± 0.050 to 3.152 ± 0.040 g (n = 3; Figure S1d,e). A simple mass‐balance estimate gives approximately 8–14 mg nominal PVA carryover per 3.0 g EGaIn (approximately 0.25–0.47 wt% relative to EGaIn). Because the recovered mass still contains residual solvent, this range is an operational estimate rather than a direct chemical measurement of surface‐bound PVA.

To more clearly resolve the PVA/oxide interfacial structure, we added magnified TEM images with explicit layer annotations and scanning transmission electron microscopy (STEM)‐EDS line‐profile analysis in Figure S3. The annotated TEM images show that the PmLMP interface consists of an outer PVA‐derived organic layer and an underlying native oxide layer (Figure S3a,b). STEM‐EDS mapping further confirms that the carbon signal associated with PVA is localized at the particle periphery, while the oxygen signal is concentrated near the oxidized liquid‐metal surface (Figure S3c,d). The corresponding EDS line profile across the interface shows a carbon‐rich outer region followed by a region in which O and Ga signals increase together, supporting the sequential arrangement of the PVA‐rich organic layer on top of the native Ga_2_O_3_ oxide shell (Figure S3e). Based on the combined TEM contrast and EDS line‐profile analysis, the PVA‐derived organic layer was estimated to be approximately 7–10 nm thick, whereas the underlying native oxide shell was estimated to be approximately 3–5 nm thick. This interfacial interpretation is consistent with reported interactions between PVA hydroxyl groups and oxide‐bearing liquid‐metal surfaces [33]. Because the spray‐printing performance is governed by the formulation‐dependent particle interface, we next examined how PVA‐shell thickness affects film microstructure, electrical transport, adhesion, and deformation‐related morphology before device fabrication. Cross‐sectional SEM imaging revealed a pronounced difference in the microstructures formed by PVA_LMP and PmLMP films (Figure 2d). The PVA_LMP coating exhibited a highly irregular and discontinuous morphology, attributable to the presence of an excessively thick PVA layer. This excessive PVA layer hindered direct particle‐particle contact, resulting in isolated liquid metal domains rather than a continuous conductive network. Consequently, the PVA_LMP film exhibited substantially reduced electrical conductivity (0.19 × 10^6^ S m^−^^1^), which was approximately eightfold lower than that of the PmLMP film (1.5 × 10^6^ S m^−^^1^) (Figure 2e). The change in surface chemical composition before and after PVA modification was analyzed by Fourier transform infrared spectroscopy (FT‐IR) (Figure 2f). Both PVA_LMP and PmLMP exhibited characteristic absorption bands corresponding to the PVA chains. Prominent peaks were observed at 3380 cm^−1^ (O─H stretching), 2940 cm^−1^ (C‐H stretching), and 1645 cm^−1^ (bound water‐related O─H bending), consistent with the spectral features of PVA [52, 53, 54]. A recent study on hydroxyl‐rich PVA coating‐mediated adhesion of liquid metals further supports the role of PVA hydroxyl groups in interfacial interactions with oxide‐rich gallium‐based liquid‐metal surfaces [55]. To provide additional support for this interfacial interpretation, we performed density functional theory (DFT) calculations as a molecular‐level feasibility check using isopropanol as a model compound for the PVA repeat unit and Ga(OH)_3_ as a hydroxylated Ga_2_O_3_ surface‐site model (Figure S4). The optimized model showed a geometrically plausible O─H···O hydrogen‐bonding configuration between the PVA hydroxyl group and the hydroxylated gallium oxide site. The calculated interaction energy was in the range of a moderate hydrogen bond, and noncovalent interaction (NCI) analysis showed an attractive interaction feature along the O–H···O axis. Together with the literature precedent on hydroxyl‐rich PVA coating‐mediated adhesion at liquid‐metal oxide interfaces, these results provide supporting evidence for possible PVA–Ga_2_O_3_ hydrogen‐bonding interactions, although the FT‐IR spectra alone do not directly resolve this interaction. Zeta potential measurements provided insights into the mechanism of immediate self‐fusing. The pristine DI_LMP exhibited a highly negative potential of −70.1 mV, creating a strong electrostatic barrier that prevents particle contact [56, 57]. Following PVA modification, the surface potential was significantly reduced to approximately −38 mV for PmLMP (Figure 2g). This reduction in surface charge is critical not only for maintaining colloidal stability, but also for lowering the electrostatic energy barrier that separates neighboring particles. Under the hydrodynamic forces applied during the spray‐printing process, the weakened electrostatic repulsion allows PmLMP particles to approach intimate contact distances *(H ≈ 0)* [58, 59]. Recent capillary‐dominant work combined PVA‐enhanced liquid‐bridge attraction with citric‐acid‐mediated oxide thinning, which reduced both shell thickness and rupture resistance [60]. The rupture values reported by Zhou et al. (3.48–3.15 µN) and 266.08 nN yield force measured here by atomic force microscopy (AFM) are not directly comparable because the particle dimensions, probe/contact geometries, loading modes, and rupture criteria differ; the comparison is mechanistic rather than a ranking of absolute shell strength. In contrast, capillary attraction alone is insufficient to rupture the chemically intact PVA/native‐oxide shells used here. High‐velocity spray impact provides the primary activation, after which capillary and Marangoni flows promote close contact, neck growth, and network consolidation. Adhesion measurements using a rheometer revealed clear differences among the formulations (Figure 2h). PVA_LMP showed the highest adhesion, consistent with its excessively thick PVA layer. In contrast, PmLMP exhibited lower adhesion than PVA_LMP but remained more adhesive than pristine LMP. Importantly, the adhesion of PmLMP was sufficient to maintain stable conductive traces on soft substrates, enabling robust circuit formation. Contact‐angle (CA) measurements on various elastomeric substrates showed that PmLMP exhibited average contact angles of 69.4° on thermoplastic polyurethane (TPU), 77.4° on polydimethylsiloxane (PDMS), and 83.7° on Ecoflex (Figure 2i; Figure S5a,b). To quantitatively analyze the surface energy modification, we calculated the liquid surface tension γ_LV_ of PmLMP using the equation of state for interfacial tensions [61, 62, 63, 64]:

(1) $$\cos\theta =-1+2\sqrt{\frac{\gamma_{\mathrm{SV}}}{\gamma_{\mathrm{LV}}}}\;e^{-\beta {\left(\gamma_{\mathrm{LV}}-\gamma_{\mathrm{SV}}\right)}^{2}}\;$$

where θ is the measured contact angle, γ_SV_ is the surface energy of the solid substrate, and β is a constant (typically 1.24 × 10^−4^ (mJ/m^2^)^−^^2^) [64]. By substituting the known surface energy values of the substrates and the measured contact angles into this equation, the derived surface tension of PmLMP was determined to be approximately 54.6 mN m^−^^1^ (Figure S5c). The value is lower than the contact‐angle‐derived value of approximately 72.8 mN m^−^^1^ for DI_LMP obtained using the same analysis (Figure S5c). Reported interfacial tensions near 500 mN m^−^^1^ for bulk gallium‐based liquid metals refer to different measurement conditions [65, 66]. Accordingly, the value reported here is treated as an effective ink property rather than a direct measurement of the intrinsic surface tension of EGaIn. The lower effective value supports improved wetting and interfacial contact. Pristine LMPs, when sprayed onto a soft substrate, readily coalesced into large droplets due to their low adhesion and dominant surface‐tension‐driven mobility. Consequently, the printed network collapsed into a discontinuous morphology under stretching (Figure 2j(i)). In contrast, PVA_LMP exhibited a different morphology. Despite its high wettability toward the substrate, the excessively thick PVA coating limited effective metallic contact and promoted crack formation during stretching (Figure 2j(ii)). By comparison, PmLMP retained a continuous and deformable particle network, reflecting balanced adhesion and controlled polymer coating (Figure 2j(iii)). Consistently, as shown in Figure S6, both pristine LMP and PVA_LMP films exhibited pronounced cracking or phase separation under strain, whereas PmLMP films preserved continuous pathways. Cross‐sectional SEM analysis further examined the structural origin of this morphology (Figure 2k). Since the spray‐printed morphology involves a complex intermingling of components, we first examined drop‐cast samples to clearly visualize the interface. The false‐colored SEM images reveal PVA fibrils (light blue) stretching between the PmLMP particles (red) and the substrate, acting as adhesive bridges. This observation is consistent with the adhesion mechanism proposed in Figure 1c. Furthermore, in the actual spray‐printed PmLMP films, the PVA fibrils formed a web‐like network bridging neighboring necked PmLMP particles (red) (Figure 2l). This morphology supports the proposed role of PVA chains in mediating particle bridging and network stabilization during PmLMP film formation (Figure 1d; Figure S7). Cross‐sectional SEM analysis of the control formulations further clarified the morphological differences among DI_LMP, PVA_LMP, and PmLMP (Figures S8 and S9). DI_LMP showed loosely stacked or aggregated liquid‐metal domains with limited substrate adhesion, whereas PVA_LMP exhibited PVA‐rich web‐like structures that can restrict metallic contact. In contrast, PmLMP retained a thinner PVA interfacial network that supports particle bridging and adhesion while allowing metallic necking between neighboring particles.

### 2.3 Impact‐Driven Particle Rupture and Conductivity Activation

To identify a deposition method capable of activating PmLMP self‐fusion, printing strategies imposing different mechanical stimuli were compared (Figure 3a). In screen printing, the applied shear stress is insufficient to disrupt the oxide‐polymer shell of PmLMP, leaving particles electrically isolated after deposition. In contrast, spray deposition delivers aerosolized PmLMPs at high velocity, generating localized impact forces upon substrate contact that rupture the shell and enable immediate particle fusion and conductive pathway formation (electrical percolation). Unlike conventional approaches that require a separate activation step after printing, our process relies on high‐velocity impact during deposition. This impact immediately ruptures the oxide shell, exposing fresh metallic Ga surfaces and enabling direct particle–particle fusion upon contact. As a result, conductive networks are formed without additional post‐processing. Screen‐printed films exhibit a morphology dominated by non‐ruptured particles, where the intact insulating oxide shells prevent the formation of continuous conductive pathways. In contrast, spray‐printed films show extensive particle rupture, particularly near the substrate interface (Figure 3b). This rupture leads to particle collapse and the formation of a denser, highly interconnected network. Chemical analysis using X‐ray Photoelectron Spectroscopy (XPS) was performed to chemically verify the mechanically induced rupture of the oxide shell. In screen‐printed films, the spectra are dominated by Ga^3^^+^ signals associated with the surface oxide (Ga_2_O_3_), indicating that the metallic cores remain encapsulated. Crucially, the spray‐printed films revealed the emergence of distinct In peaks, which were negligible in the screen‐printed counterparts [65, 66]. This observation can be explained by the preferential oxidation behavior of Ga. Because Ga has a lower reduction potential than In, it oxidizes more readily, forming a surface‐segregated Ga_2_O_3_ shell that encapsulates and effectively shields the in enriched metallic core from surface‐sensitive XPS detection. Consequently, the appearance of strong In signals together with the recovery of metallic Ga^0^ features in the spray‐printed films provides chemical evidence consistent with oxide‐shell rupture (Figure 3c). To evaluate the mechanical influence of the PVA protective layer on rupture dynamics, AFM nanoindentation was performed on individual PmLMPs (Figure 3d) [67, 68, 69]. A key engineering consideration is whether the additional polymer coating excessively reinforces the particle, potentially impeding impact‐driven activation. To address this concern, the experimentally measured yield force was benchmarked against reported rupture forces of bare liquid metal particles stabilized solely by their native oxide shell. The force–distance curve showed an initial increase in repulsive force upon tip–particle contact, followed by a distinct yield point at 266.08 nN (Figure 3e). This value is comparable to, or only slightly higher than, the reported threshold for bare particles (200–250 nN) [68], indicating that the ultrathin PVA shell does not substantially suppress the mechanical rupturability of the particle. After the yield point, the AFM tip continued to penetrate the particle until reaching the underlying substrate, consistent with failure of the oxide/PVA shell and collapse of the liquid‐metal core. During retraction, the curve showed pronounced hysteresis and a pull‐off force of approximately −100 nN, consistent with the adhesive and viscoelastic nature of the PVA‐modified interface. To further correlate the force–distance response with morphological changes, AFM 3D topography was acquired before and after indentation (Figure 3f). The post‐indentation morphology showed localized collapse at the indentation site, supporting the assignment of the measured yield point to shell rupture rather than simple elastic deformation. Together with the XPS results showing the emergence of metallic Ga^0^ and In signals after spray deposition, these results support impact‐induced rupture of the oxide/PVA shell as the primary activation mechanism for immediate electrical percolation. The AFM‐derived rupture threshold therefore serves as the mechanical activation criterion for the subsequent spray‐deposition study, where nozzle geometry, air‐to‐liquid ratio, gas pressure, and working distance are optimized to provide sufficient impact energy for shell rupture. The AFM‐measured rupture energy corresponds to a critical impact velocity of only ∼4 m s^−^^1^, which is well below the conservative spray‐particle velocity estimate under the optimized 30 psi condition; a quantitative kinetic‐energy‐vs.‐critical‐rupture‐energy (E_k_ vs E_c_) comparison is provided in Note S1 and is consistent with the pressure‐ and distance‐dependent conductivity trends in Figure 3k,l. To enable direct self‐fusion printing of PmLMPs, we employed a flow‐controllable airbrush system and systematically investigated how nozzle geometry and flow modulation govern particle fusion behavior [43, 67]. As illustrated in Figure 3g, the needle retraction distance in the airbrush directly controls the effective nozzle aperture (*A*). By adjusting the needle position, we effectively modulated the ink discharge rate, thereby controlling the Air‐to‐Liquid Ratio (ALR) of the atomized jet [70, 71, 72, 73]. This flow regulation directly dictates the acceleration mechanism of the in‐flight particles. Under high ALR conditions (i.e., minimal needle retraction and low *A*), the mass loading of the ink on the airflow is minimized. Consequently, the kinetic energy of the high‐pressure air is concentrated on a smaller mass of particles, accelerating them to a high terminal velocity *v*. Conversely, a large needle retraction results in a low ALR with increased mass loading, which diminishes the aerodynamic acceleration efficiency, causing particles to reach the substrate at lower velocities. The physical state of the particles upon impact is governed by the interplay between their kinetic energy *E_k_* =  1/2 *mv* ^2^ and the critical rupture energy *E* *crit* of the protective shell. As depicted in Figure 3h, particles accelerated under high ALR conditions achieve kinetic energies exceeding the critical threshold (*E_k_* > *E*_crit_). Upon impact, the resulting compressive stress induces dynamic oxide rupture, fracturing both the native gallium oxide skin and the PVA shell to expose the liquid metal core and establish instantaneous conductive pathways. In contrast, under low ALR conditions, the particle kinetic energy remains below the critical threshold. In this regime, particles retain their spherical morphology, while hydrodynamic cushioning dissipates impact stress, preserving the insulating oxide shell and preventing electrical percolation. Experimental validation of this energy criterion is provided by the morphological analyses of printed patterns shown in Figure S10 and Videos S1 and S2. Based on this mechano‐fluidic mechanism, we systematically optimized the spray and sonication parameters to maximize electrical conductivity (Figure 3i). Particle size distribution, controlled by ultrasonic amplitude during ink formulation, plays a critical role in the printing performance. Typically, excessively large particles suffer from limited acceleration due to high inertia, while particles that are too small lack sufficient kinetic energy for rupture [74, 75, 76]. Initially, we hypothesized that reducing particle size by increasing the ultrasonic amplitude would be advantageous for high‐resolution printing [51, 77]. However, despite rigorous cooling, high‐amplitude sonication introduced excessive energy into the aqueous medium, leading to a pronounced temperature rise and promoting the formation of Ga/O‐rich oxide‐ or hydroxide‐related byproducts (Figure S11) [78]. Rather than mechanically reinforcing the particles, these non‐metallic byproducts can interfere with effective liquid‐metal contact and particle–particle coalescence. Consequently, samples prepared with amplitudes exceeding 50% exhibited a sharp decrease in conductivity. Electron microscopy and EDS analysis of the high‐amplitude samples support this interpretation: the 50% amplitude sample showed irregular surface deposits and aggregated features, while the 100% amplitude sample exhibited Ga‐ and O‐rich structures with negligible In signal, consistent with oxide/hydroxide‐rich byproduct formation rather than smooth liquid‐metal particles (Figure S12). We therefore identified an optimal amplitude of 20%, which provided the highest conductivity without substantial byproduct formation (Figure 3j). The measured mean particle diameter of the optimized DI water/ethanol (3:1 v/v) PmLMP formulation was 1182.9 nm (Figure 4i); this value, rather than the 309.7 nm DI‐only control or the illustrative R = 250 nm capillary‐model radius in Figure 4h, was used as the representative diameter in the impact‐energy calculation in Note S1. The carrier gas pressure was identified as another critical driving factor. Increasing the compressor pressure increased the particle flight velocity, thereby elevating the kinetic energy *E* _*k*_ above the critical rupture threshold *E* _*Crit*_. Experimentally, electrical conductivity saturated at pressures above 30 psi, indicating that sufficiently high‐pressure conditions are required for effective shell rupture during spray deposition (Figure 3k). Crucially, the spray working distance (height) serves as a key parameter governing both solvent evaporation and kinetic energy retention during deposition (Figure 3l). Because the PmLMP ink is formulated in a DI water/ethanol (3:1 v/v) binary solvent system, sufficient flight time is required to allow partial solvent evaporation before particle impact. When the nozzle is positioned too close to the substrate, insufficient evaporation results in a wet film, which mechanically cushions particle impact and suppresses impact‐induced fusion. At the tested 6 cm working distance, the increased flight distance led to significant kinetic energy dissipation due to air resistance, reducing the impact energy below the critical threshold (*E_k_* < *E*_crit_) and causing a sharp decline in conductivity. An optimal working distance of 3 cm was therefore identified, at which balanced solvent evaporation and sufficient impact energy enable efficient oxide rupture and conductive network formation. To probe the contributions of solvent composition and evaporation conditions separately from the impact‐related variables, we varied the DI water/ethanol ratio from 1:1 to 4:1 and the substrate temperature from RT to 100°C while holding the nozzle geometry, carrier‐gas pressure, working distance, needle setting, and number of spray passes constant (Figure S13). Conductivity increased only modestly with substrate temperature, consistent with accelerated solvent evaporation, whereas the 3:1 formulation already achieved high conductivity at RT. We therefore selected the 3:1 formulation at RT as the general printing condition for compatibility with temperature‐sensitive substrates; heating serves as an optional process aid rather than a required post‐treatment. Because the liquid‐delivery rate was not independently metered, this matrix is interpreted as a solvent‐composition/temperature study rather than a complete response‐surface optimization.

**FIGURE 3:**
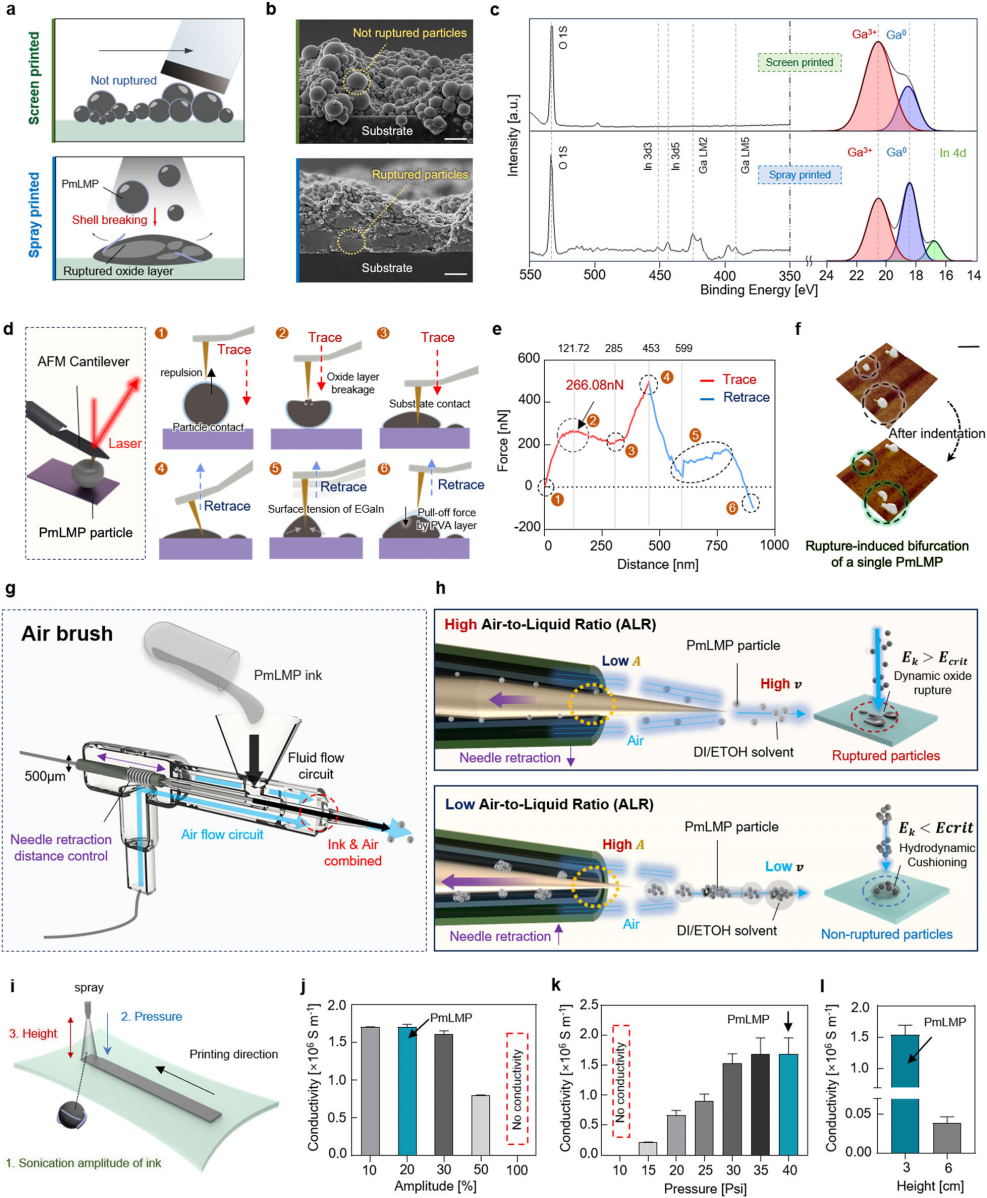
Mechano‐fluidic activation mechanism and process optimization of spray‐printed PmLMPs. a Schematic comparison of the deposition dynamics between screen printing and spray printing. b Cross‐sectional SEM images showing the morphological difference between the screen‐printed film and spray‐printed film. c XPS spectra (Ga 3d and O 1s regions) confirming the exposure of metallic Ga (Ga^0^) in the spray‐printed layer due to oxide rupture. d Schematic illustration of the AFM nano‐indentation process on a single PmLMP to evaluate shell mechanics. e Force‐distance curve revealing the oxide rupture point, substrate contact, and viscoelastic pull‐off force. f AFM 3D topography images before and after indentation. g Schematic of the flow‐controllable airbrush system highlighting the needle retraction mechanism for ALR modulation. h Proposed sintering mechanism: High ALR leads to dynamic oxide rupture (*E* _*k*_  > *E* _*Crit*_) while Low ALR results in hydrodynamic cushioning (*E* _*k*_  < *E* _*Crit*_). i Schematic of the key process parameters: particle size, pressure, and height. (j–l) Electrical conductivity as a function of ultrasonic amplitude (j), carrier‐gas pressure (k), and spray height (l) (n = 4). The observed trends are consistent with the impact‐driven activation model. *Data in j–l are presented as mean ± SD (n = 4 per condition); error bars indicate SD. No inferential comparison is reported for these panels*.

**FIGURE 4:**
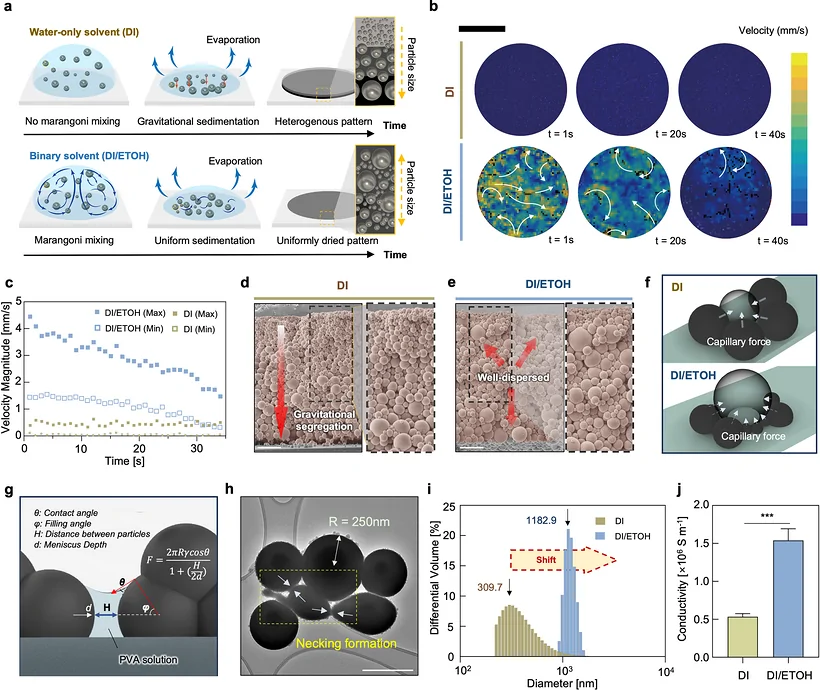
Hydrodynamic‐Assisted Particle Fusion via Marangoni and Capillary Forces. a Schematic illustration of Marangoni convection induced by surface‐tension gradients in the binary solvent system. b High‐speed visualization of internal flow dynamics using fluorescent tracer particles (n = 3). c Particle Image Velocimetry (PIV) analysis comparing flow velocities between single (DI) and binary (DI water/ethanol) solvent systems (n = 3). d Cross‐sectional SEM image of the control film (DI) showing gravitational segregation. e Cross‐sectional SEM image of the optimized film (binary solvent) exhibiting interstitial filling and dense packing. f Schematic illustration of the capillary‐bridge model. g Governing equation for the lateral capillary force F exerted between particles during drying. h TEM image showing neck formation at particle contacts. i Particle‐size distributions of the DI‐only control and optimized DI water/ethanol (3:1 v/v) PmLMP formulation, with mean diameters of 309.7 and 1182.9 nm, respectively (n = 3). j Electrical‐conductivity comparison showing a threefold enhancement in the binary‐solvent system (n = 5). Data in j are presented as mean ± SD (n = 5 conductivity measurements per solvent condition); error bars indicate SD. The single‐ vs. binary‐solvent comparison in j was analyzed using one‐way ANOVA. ***p < 0.001.

Under these optimized conditions, we next examined the effect of repeated spray cycles using top‐view SEM imaging. The SEM images reveal that particles indeed rupture, followed by leakage and spreading on the substrate. With increasing spray cycles, a denser and more continuous conductive network is formed (Figure S14).

### 2.4 Hydrodynamic‐Assisted Particle Fusion via Marangoni and Capillary Forces

Although the initial impact‐induced fusion establishes electrical percolation, a fraction of particles can remain encapsulated within intact oxide shells due to insufficient collision energy. The hydrodynamic process discussed in this section is therefore treated as a secondary consolidation step that acts after impact‐induced activation, rather than as a stand‐alone alternative to spray deposition. Drop‐cast samples were used here as a model drying geometry to clearly visualize Marangoni‐driven particle redistribution and capillary necking, which are difficult to resolve during high‐speed spray impact. To further rupture these residual shells and enhance interparticle interconnection within the conductive network, we employed a hydrodynamic‐assisted fusion strategy that leverages Marangoni and capillary forces during the drying process. By replacing a single‐solvent system (DI water) with a binary solvent mixture (DI water/ethanol) possessing distinct surface tensions and evaporation rates, a surface‐tension gradient is generated within the drying droplet. This gradient drives Marangoni convection, which redistributes particles, promotes additional interparticle contacts, and facilitates flow‐mediated consolidation of the conductive network (Figure 4a) [79]. These Marangoni‐driven vortical flows were observed to mitigate gravitational sedimentation and promote homogeneous particle mixing, thereby increasing interfacial contact area and enhancing capillary‐driven consolidation during drying. To directly visualize these flow dynamics, fluorescent tracer particles were introduced and tracked using high‐speed imaging (Figure 4b). While the single‐solvent system remained nearly static, the binary solvent system exhibited dynamic vortical flows. Particle Image Velocimetry (PIV) analysis revealed that the binary solvent induced flow velocities up to 50 times faster (average 22.4‐fold increase) compared to the single‐solvent system (Figure 4c; Videos S3 and S4). To further compare drying behavior, we monitored the time‐dependent weight change of drop‐cast samples using a precision balance. The DI water/ethanol binary solvent exhibited a faster mass loss than the DI‐only control (Figure S15). This faster drying is primarily attributed to the higher volatility of ethanol in the binary solvent system, rather than to Marangoni convection alone. Therefore, in this work, Marangoni flow is interpreted mainly as a particle‐redistribution mechanism, as supported by PIV analysis, while the increased evaporation rate is attributed mainly to the solvent composition (Figure S15). Cross‐sectional SEM analysis demonstrated the structural impact of these fluid dynamics. The DI control suffered from gravitational segregation, causing particles to stratify by size. This stacking of similar‐sized particles tends to form broad‐area contacts, where the capillary force is distributed over a larger effective contact area, thereby suppressing localized stress concentration (Figure 4d, Figure S16). In contrast, the Marangoni convection in the binary system promoted interstitial filling, where smaller particles were dynamically wedged into the voids between larger ones (Figure 4e). This heterogeneous mixing increased the coordination number and, more importantly, generated nanoscale point‐contact geometries between dissimilarly sized particles, where the local stress can be concentrated due to the small effective contact area (Figure 4f). The observed densification can be quantitatively explained by the lateral capillary force *F* exerted during drying, governed by the equation presented in Figure 4g [80, 81].

(2) $$F=\frac{2\pi R\gamma cos\theta }{1+\left(\frac{H}{2d}\right)}$$

We identified that the PVA surface modification of PmLMP plays a pivotal role in optimizing the variables of this equation to maximize liquid metal particle‐fusion efficiency. First, the PVA coating mitigates electrostatic repulsion, minimizing the inter‐particle distance *H*. Unlike the control group (DI_LMP), which exhibited a high zeta potential of −70 mV causing strong repulsion, PmLMP showed a reduced potential of −38 mV, effectively reducing the interparticle separation *H*. According to Equation (2), decreasing H reduces the denominator and increases the capillary force, allowing it to approach a finite maximum under intimate‐contact conditions. Second, the hydrophilicity of PVA enhances surface wettability. The reduced contact angle on various substrates (Figure 2i) increases the *cos*θ term in the governing equation, contributing to stronger capillary attraction. Furthermore, the enhanced adhesion of PmLMP (Figure 2h) acts as a binder, preventing elastic rebound of the particle network during rapid capillary retraction. To evaluate whether the estimated capillary force could plausibly contribute to secondary shell rupture, we compared the capillary‐force estimate with the mechanical yield threshold determined via AFM nanoindentation in “Impact‐Driven Particle Rupture and Conductivity Activation”. As previously established, a single PmLMP requires an external load of 266.08 nN to mechanically rupture its oxide shell. Our theoretical model, assuming intimate particle contact (H ≈ 0) driven by the removal of electrostatic repulsion, predicts a lateral capillary force of ∼79 nN at an individual interparticle meniscus. We emphasize that this value should not be interpreted as a single external load distributed over multiple neighboring particles. Rather, each capillary bridge acts locally at its own particle–particle junction. Thus, increasing the coordination number does not increase the stress at a given contact; instead, it increases the number of independent potential fusion sites throughout the network.

The key factor for local shell rupture is the effective contact area. Marangoni‐assisted interstitial filling creates nanoscale point contacts between dissimilarly sized particles, where the local stress can be approximated as σ ≈ F/A. Using an explicitly illustrative circular contact radius of ∼11 nm, a 79 nN capillary force gives a nominal local stress of ∼200 MPa. Because the transient contact area was not measured, this calculation is presented only as a sensitivity/plausibility estimate and is not treated as a direct stress measurement or independent proof of shell failure. Broader contacts would distribute the same capillary force over a larger area and therefore produce lower local stress. Thus, the capillary force is not amplified by multiple contacts; point‐contact geometry can concentrate local stress, while an increased coordination number increases the number of potential fusion sites and promotes network densification. This self‐fusion phenomenon was supported by transmission electron microscopy (TEM), which captured neck formation at particle contacts indicating the formation of a fused alloy structure (Figure 4h). In this model drying configuration, coalescence and neck formation occurred without high‐temperature annealing or mechanical pressing, indicating that solvent‐driven hydrodynamic forces can further consolidate particles after the initial impact‐induced activation step. Thus, Figure 4 is intended to visualize the secondary Marangoni/capillary fusion process rather than to present drop‐casting as an alternative fabrication route. Particle‐size analysis using a particle size and zeta‐potential analyzer gave mean diameters of 309.7 nm for the DI‐only control and 1182.9 nm for the optimized DI water/ethanol (3:1 v/v) formulation (Figure 4i) [38]. The larger mean diameter in the optimized binary‐solvent formulation is consistent with secondary particle coalescence during drying, which extends conductive pathways and yields a conductivity of 1.5 × 10^6^ S m^−^^1^, a threefold enhancement over the control (Figure 4j).

### 2.5 High‐Resolution Patterning and Electromechanical Robustness of PmLMP Conductors

Having established the particle interface and self‐fusion mechanism, we next evaluated whether the optimized spray‐printing process could provide both microscale pattern fidelity and reproducible electrical performance. Laser‐confocal profiles revealed variations in height and cross‐sectional geometry under the present mask‐assisted printing conditions (Figure 5a, Figure S17a–d), indicating limitations in dimensional fidelity and thickness uniformity. Nevertheless, resistance measurements confirmed electrical conduction down to a nominal linewidth of 50 µm (Figure S17e). These results demonstrate the feasibility of conductive microscale patterning, but do not establish equivalent conductivity or local fusion efficiency across linewidths. A 4 × 4 array printed under identical conditions exhibited an average conductivity of 1.55 × 10^6^ S m^−^^1^ with limited pixel‐to‐pixel variation (Figure 5b), demonstrating spatial reproducibility within the measured array.

**FIGURE 5:**
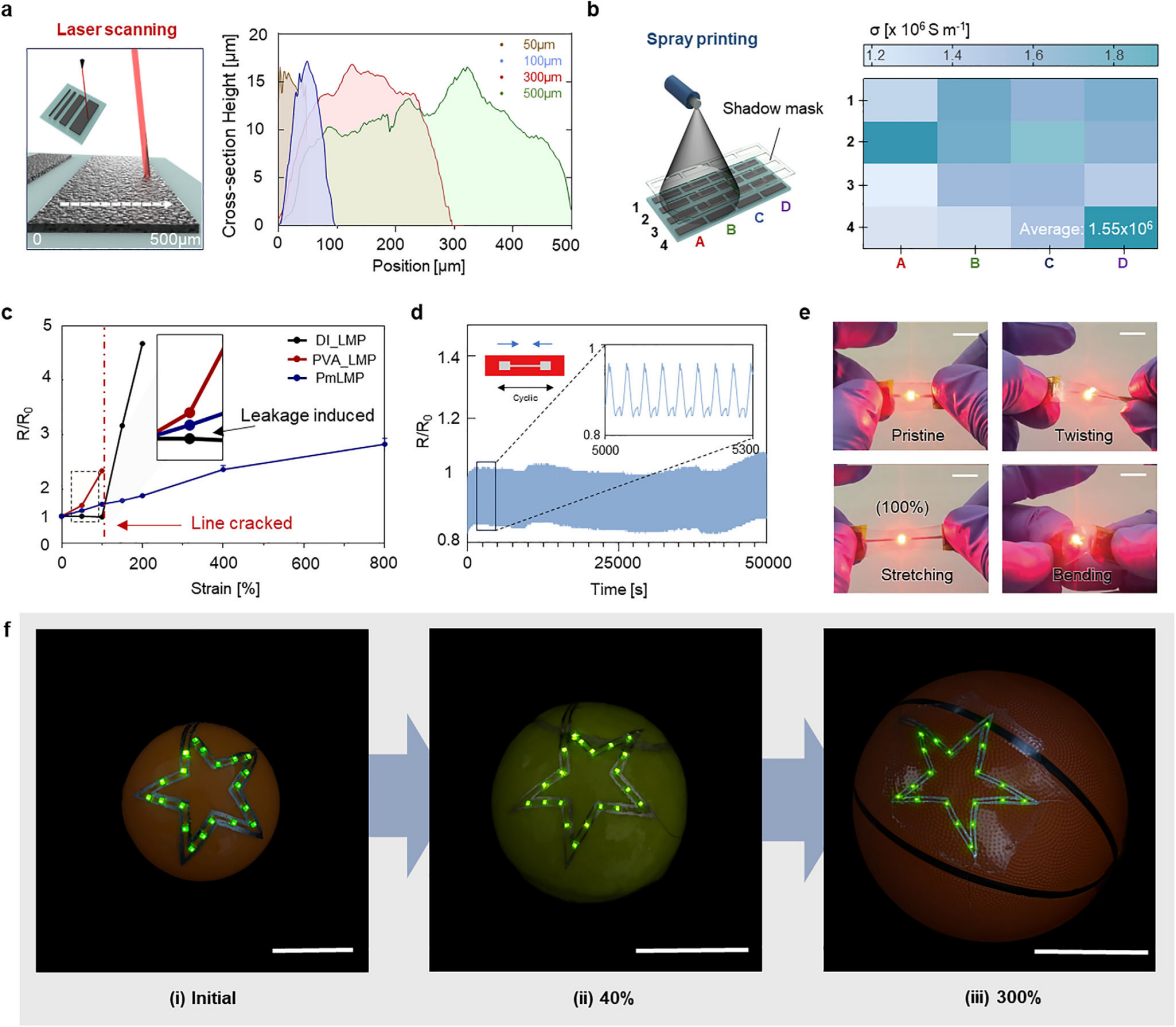
Scalable printing and electromechanical performance of spray‐printed PmLMP conductors. a Laser‐scanning microscopy and cross‐sectional height profiles of nominal 50, 100, 300, and 500 µm traces. b Conductivity map of a batch‐printed 4 × 4 array (average, 1.55 × 10^6^ S m^−^^1^). c Relative resistance (R/R_0_) vs. strain for DI_LMP, PVA_LMP, and PmLMP traces (n = 5). d Cyclic‐stretching response of PmLMP at 100% strain over 50,000 s, corresponding to approximately 1,400 stretch‐release cycles at a period of ∼36 s (∼0.028 Hz) (n = 3); inset, representative cycles between 5,000 and 5,300 s. e Operating LED circuit in the pristine, twisting, 100%‐stretching, and bending states. f Operational star‐shaped LED arrays separately prepared on (i) table‐tennis, (ii) tennis, and (iii) basketball surfaces. These images demonstrate qualitative conformability and are not strain measurements. Scale bars: 2, 3, and 10 cm, respectively. *In c, data are presented as mean ± SD (n = 5 per formulation); error bars indicate SD. No inferential comparisons are reported for b–d*.

Quantitative electromechanical performance was evaluated under monotonic and cyclic loading (Figure 5c,d). Under monotonic tension, DI_LMP showed a steep resistance increase associated with leakage‐induced loss of continuity, whereas the over‐coated PVA_LMP trace fractured near 100% strain. In contrast, PmLMP retained electrical continuity beyond 200% and up to 800% strain, with R/R_0_ increasing only gradually to approximately 2.8 (Figure 5c; n = 5). The PmLMP conductor also showed reproducible resistance modulation with limited drift during cyclic stretching at 100% strain over 50,000 s, corresponding to approximately 1,400 continuous stretch‐release cycles (Figure 5d; n = 3). Consistent with these measurements, an integrated light‐emitting diode (LED) remained illuminated in the pristine, twisting, 100% stretching, and bending states (Figure 5e). Separately prepared star‐shaped LED arrays also remained operational after placement on table‐tennis, tennis, and basketball surfaces of different diameter and curvature (Figure 5f). These photographs are treated only as qualitative conformability demonstrations, not as strain measurements. The resistance–strain and cyclic‐loading data in Figure 5c,d provide quantitative electromechanical evidence, while Figure 5e demonstrates LED operation under the specified deformations, including 100% stretching. Complementary conductivity benchmarks, processing‐window, and substrate‐comparison data are provided in Figure S18. On nonpolar PDMS and Ecoflex, the untreated surfaces provide few polar functional groups for hydrogen‐bonding interactions with PVA, resulting in relatively weak interfacial interaction with the PVA‐modified particles. Oxygen‐plasma pretreatment increases the surface polarity and thereby promotes more favorable interfacial interactions, producing visibly more uniform and continuous printed traces during manual stretching than on untreated substrates (Figure S19). Because this comparison was qualitative, it is discussed as a substrate‐preparation observation rather than a quantitative adhesion measurement. These observations do not establish substrate‐specific cyclic durability on PDMS or Ecoflex.

### 2.6 Integrated Application Demonstrations of PmLMP‐Based Soft Electronics

Having established quantitative electromechanical performance in Figure 5, we next evaluated representative device‐level applications in a unified sequence spanning automated assembly, wound treatment, large‐area textile patterning, and wearable electrophysiology (Figure 6). The immediately conductive PmLMP interconnects enabled automated pick‐and‐place assembly of a microcontroller unit (MCU), resistor, and red LED directly on the as‐printed flexible circuit; the assembled device remained functional after folding (Figure 6a). Additional chip‐programming, large‐area LED‐control‐array, and device‐stretching demonstrations are provided in Figure S20.

**FIGURE 6:**
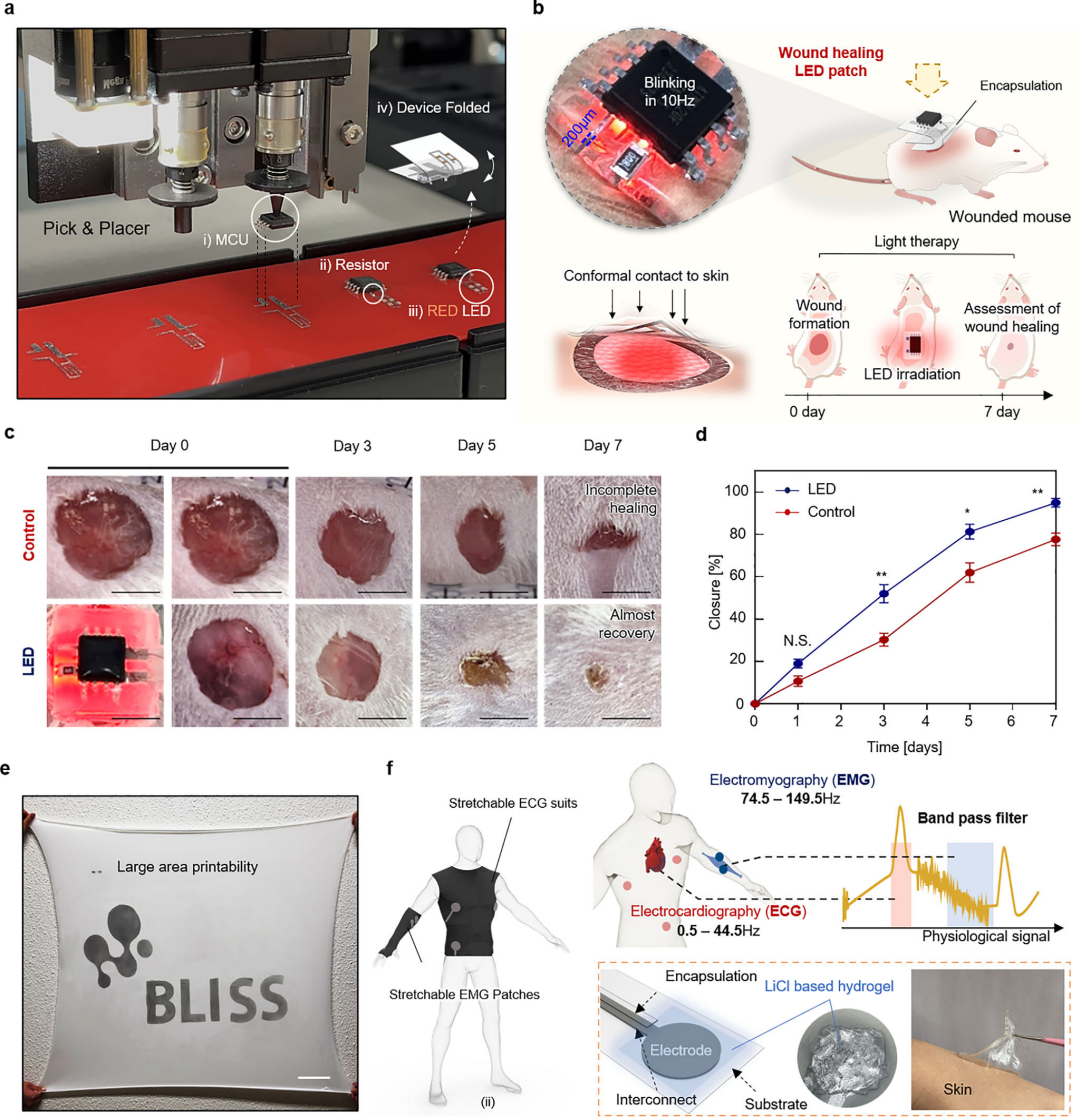
Integrated PmLMP soft‐electronic applications. a Automated pick‐and‐place assembly of a microcontroller unit (MCU), resistor, and red light‐emitting diode (LED) on printed interconnects; inset, folded device. b Wound‐healing LED patch and 7‐day irradiation protocol. c Full‐thickness wounds in control and LED‐treated mice from day 0 to day 7 (scale bars = 0.5 cm). d Wound closure (n = 4). e Textile patterning (scale bar = 10 cm). f Wearable electrocardiography/electromyography (ECG/EMG) platform, signal bands, and the PmLMP‐electrode/LiCl‐hydrogel interface. Data in d are presented as mean ± SD (n = 4 animals per group); error bars indicate SD. Wound closure was analyzed using two‐way ANOVA with treatment group and time as factors, followed by Tukey's multiple‐comparisons test. *p < 0.05, **p < 0.01; N.S., not significant.

For a representative biomedical application, a flexible wound‐healing patch incorporating 0402 red LEDs (640 nm) was operated at 10 Hz and conformed to the wound surface (Figure 6b). In a murine full‐thickness excisional‐wound model, the LED‐treated group showed accelerated macroscopic closure relative to the untreated control over 7 days (Figure 6c,d; n = 4). Patch fabrication and deformation are shown in Figure S21, while the detailed histological and immunohistochemical analyses are retained in Figure S22.

Large‐area scalability was further demonstrated by printing a complex conductive pattern on a textile banner (Figure 6e). The same platform was integrated with a LiCl‐hydrogel skin interface for wearable EMG and ECG acquisition (Figure 6f). Detailed EMG/ECG waveforms and signal‐to‐noise comparisons are provided in Figure S23, and the wireless EMG‐based human–machine interface and ECG‐monitoring workflows are provided in Figure S24 and Videos S5 and S6. Together, these results extend the quantitative material validation in Figure 5 to device‐level applications spanning biomedical, textile, and wearable platforms.

## 3 Discussion

In this work, we address key processing limitations of liquid‐metal conductors by combining PVA surface modification with spray deposition, thereby improving wettability and enabling immediate electrical percolation during deposition. Impact‐induced self‐fusion enables in situ formation of conductive networks. Marangoni‐ and capillary‐assisted consolidation further stabilizes these networks, eliminating separate post‐deposition activation or post‐treatment steps and enabling scalable fabrication of immediately conductive, deformable liquid‐metal conductors on soft substrates. While this impact‐induced activation pathway is supported by the present SEM, XPS, AFM, spray‐condition‐dependent and spray‐cycle‐dependent morphology, video, and electrical evidence, future high‐speed, higher‐resolution in situ or operando studies could further resolve nanoscale oxide‐cracking dynamics during spray impact. Importantly, this approach extends beyond small‐scale validation by enabling scalable, batch‐compatible fabrication through the integration of commercial off‐the‐shelf components. Using this strategy, we realized a batch‐compatible soft electronic LED patch with functional efficacy in a murine wound‐healing model. To further demonstrate practical versatility and system‐level applicability, spray‐printed PmLMP conductors were integrated into large‐area wearable bioelectronic platforms. This integration enabled real‐time electrophysiological sensing, while the wireless human‐machine interface (HMI) operation was provided as a supporting system‐level demonstration. Collectively, this mechano‐fluidic fusion approach establishes a practical materials‐processing strategy for transforming liquid metals into post‐treatment‐free, conductive, and deformable soft electronic systems. This advance opens new opportunities in wearable healthcare, bio‐integrated electronics, and human‐interactive devices.

## 4 Experimental Section

### 4.1 Fabrication of PmLMP Ink

PmLMP ink was formulated using a tip‐sonication process involving a solution of liquid metal (LM) and poly(vinyl alcohol) (PVA) with a molecular weight of 89,000–98,000 g/mol in water (Sigma‐Aldrich). First, a 5 wt% PVA solution was prepared by dissolving PVA in water. LM was synthesized by mixing gallium (Ga) and indium (In) in a 3:1 weight ratio. To produce the PmLMP ink, 3.0 g of bulk LM and 2.0 g of the prepared 5 wt% PVA aqueous solution were combined and subjected to tip sonication (Q700, Qsonica LLC) using a 1/8‐inch microtip at 20% amplitude (5 s on/off cycles) for a total of 10 min. After sonication, the dispersion was centrifuged at 2000 rpm for 3 min using a 1248R centrifuge (LaboGene), and the supernatant containing unbound or excess PVA was removed. Surface‐associated PVA was retained on the particles as the ultrathin organic interfacial layer identified by TEM/STEM‐EDS. Immediately before spray printing, the collected particles were redispersed in 1.5 mL of DI water preheated to 70°C and 0.5 mL of ethanol.

For the gravimetric estimate, three independently prepared PmLMP batches were weighed immediately before centrifugation and again after centrifugation and supernatant removal. The nominal PVA carryover was estimated from the paired mass balance using the 5 wt% stock composition under two limiting assumptions: complete retention of the nominal 3.0 g EGaIn and proportional allocation of the small pre‐weighing handling loss. Because the recovered mass contains residual solvent, the resulting 8–14 mg range is reported as an operational estimate rather than a direct assay of particle‐bound PVA. Data are reported as mean ± SD (n = 3).

### 4.2 Physicochemical Characterization of PmLMP Ink

The wetting properties and rheological behavior of the formulated PmLMP ink were investigated to evaluate its processability. The static contact angle was measured using a drop shape analyzer (SmartDrop; FEMTOBIOMED, Korea) to assess the wettability of the ink on the target substrate. To analyze the flow behavior and viscoelastic properties, rheological measurements were performed using a commercial rheometer (MCR 102; Anton Paar GmbH, Austria). Fourier transform infrared (FT‐IR) spectroscopy was performed using an INVENIO FT‐IR spectrometer (Bruker, Germany). For FT‐IR measurements, bare EGaIn, PVA aqueous solution, PVA_LMP, and PmLMP samples were prepared by drop‐casting approximately 10–20 µL of each sample onto the measurement substrate. The deposited samples were allowed to dry completely under ambient conditions to minimize oxidation. After drying, the samples were gently pressed to ensure conformal contact with the ATR crystal prior to spectral acquisition. X‐ray photoelectron spectroscopy (XPS) and ultraviolet photoelectron spectroscopy (UPS) measurements were performed using an AXIS Supra^+^ system (Kratos Analytical) to investigate the surface chemical composition and electronic structure of PmLMP thin films. Thin films were fabricated by either screen printing or spray printing under identical material formulations. All samples were prepared on identical substrates and measured under the same experimental conditions to enable direct comparison between the two printing methods. XPS was employed to analyze the elemental composition and chemical bonding states at the film surface, while UPS measurements were conducted to examine the valence band characteristics.

### 4.3 Morphological Analysis of PmLMP Ink

To observe the surface morphology of printed ink, SEM images were taken by IT‐500HR and IT‐700HR (JEOL). To obtain cross‐sectional SEM images, the printed samples were first frozen at −80°C. Pre‐cut lines made with a diamond cutter were then used as guides to split the samples in half for imaging. To verify the core–shell structure and chemical composition, transmission electron microscopy (TEM) and energy‐dispersive X‐ray spectroscopy (EDS) were performed using a JEM‐2100Plus (JEOL, Japan) operated at an acceleration voltage of 200 kV. For sample preparation, the diluted PmLMP ink was drop‐cast onto a holey carbon grid. To minimize signal interference from the carbon support film, EDS analysis was selectively conducted on particles suspended over the open holes of the grid. In addition to elemental mapping, STEM‐EDS line scan analysis was performed across individual particles to examine the spatial distribution of constituent elements. As shown in Figure 2c, the carbon (C) signal is distinctly localized to the particle shell and clearly distinguishable from the background, confirming that the detected carbon originates from the conformal PVA coating rather than the substrate.

### 4.4 Particle Size and zeta‐Potential Analysis of PVA‐Modified Liquid Metal Particles

Particle size and zeta potential measurements were performed using a particle size and zeta potential analyzer (ELS‐Z1000, Otsuka Electronics). Measurements were conducted at 25°C with a backscattering angle of 173°. The refractive index and viscosity of the dispersing media were set according to the solvent composition. Zeta potential measurements were carried out for DI_LMP, PVA_LMP, and PmLMP samples under identical conditions. For the PmLMP samples, particle size measurements were additionally performed using DI water and a DI water/ethanol mixture to compare solvent‐dependent effects associated with drying‐induced Marangoni flow. Each measurement was repeated at least three times to ensure reproducibility.

### 4.5 PmLMP Ink Spray Printing

The prepared ink was printed using an airbrush equipped with a 0.5 mm nozzle (BD‐116BK, Tool Mart Co., Ltd., Seoul, Korea) connected to an air pump (AC‐501X, Anest Iwata Sparmax Co., Ltd., Taipei, Taiwan). Unless otherwise stated, optimized printing used a carrier‐gas pressure of 30 psi and a nozzle‐to‐substrate distance of 3 cm. Patterning was achieved using a laser‐cut mask. For the solvent‐composition/temperature matrix in Figure S13, DI water/ethanol volume ratios of 1:1, 2:1, 3:1, and 4:1 and substrate temperatures of room temperature (RT), 50°C, and 100°C were evaluated by placing the substrate on a temperature‐controlled hot plate. The nozzle diameter, carrier‐gas pressure, nozzle‐to‐substrate distance, airbrush needle/liquid‐delivery setting, and number of spray passes were held constant; the liquid‐delivery rate was not independently varied in this matrix. For the nonpolar‐substrate comparison in Figure S19, PDMS and Ecoflex substrates assigned to the plasma‐treated groups were treated using an O_2_ plasma system (COVANCE; Femto Science Inc., Hwaseong, Republic of Korea) at 80 W for 3 min with an O_2_ flow rate of 50 sccm and a process pressure of 5.0 × 10^−^^1^ Torr (base pressure, 5.0 × 10^−^^2^ Torr; discharge frequency, 50 kHz; electrode setting, PE). The gas‐introduction, purge, and vent steps were each set to 30 s.

### 4.6 Particle Rupturing Force Analysis

The force‐distance (F/D) curves were obtained using an AFM (NX‐10, Park Systems, Korea). Standard contact‐mode cantilevers generally possess low spring constants, which are insufficient to exert the high range of forces (hundreds of nN to several µN) required to rupture the oxide shell within the system's deflection limits. To address this, we utilized a high‐stiffness cantilever (PPP‐NCHR, Nanosensors), typically designed for non‐contact mode applications. The measurement protocol involved a two‐step process: first, the target particle was imaged in non‐contact mode to precisely locate its apex without inducing premature deformation. Subsequently, the system was switched to contact mode at the same coordinates to perform nanoindentation and acquire F/D curves until shell rupture occurred.

### 4.7 Evaluation of Marangoni Flow

To study Marangoni flows within binary solvent droplets, micro‐particle image velocimetry (µ‐PIV) was utilized. Monodisperse fluorescent microspheres (M122074, Aladin) with a diameter of 2 µm were employed to visualize the internal flows [79]. These fluorescent particles were mixed with the ink and then deposited onto a glass substrate. The droplet's drying process was recorded using a high‐speed camera. A metal laser, coupled with the high‐speed camera, excited the fluorescent particles at a wavelength of 532 nm, while an optical filter captured the emitted fluorescence signal. Fluorescent images were recorded as video at 200 frames per second and analyzed using the PIVlab tool in Matlab to determine flow fields. Vector fields were generated through iterative 2D cross‐correlation of particle images using multiple interrogation windows. The initial window size was 64 × 64 pixels, followed by 32 × 32 pixels, with a 50% overlap in each case. This approach ensured reliable µ‐PIV results, meeting the signal‐to‐noise ratio (SNR > 3) threshold defined in PIV. The evaporation rates of PmLMP solutions prepared in deionized water (DI) and in a mixed solvent of DI and ethanol (DI + EtOH) were compared using a precision analytical balance (XSR205DUV, Mettler Toledo). A fixed volume of each solution was drop‐cast onto a substrate placed on the balance, and the mass change over time was recorded under ambient conditions until complete solvent evaporation.

### 4.8 Electrical Characterization

Electrical conductivity (𝜎) was calculated through sheet resistance measurements using a 4‐point probe. The surface resistance was calculated by measuring the voltage response to the applied current using a source meter unit (Keithley 2400, Keithley). The thickness (T), length (L), and width (W) of the samples were determined using a laser microscope (VK‐X3000, Keyence) and cross‐sectional SEM images. The electrical conductivity (𝜎) was calculated using the equation: 𝜎 = (L × I)/(W × T × V). To evaluate the relative resistance change, a force gauge, a stand with a motor, and source meter unit were used. To evaluate the long‐term electromechanical stability of the circuit printed by PmLMP ink, cyclic stretching‐relaxing tests were performed using a motorized X‐axis translation stage (X stage; Jaeil Optical Corp., Seoul, Korea). The resistance changes were monitored in real‐time while the samples were subjected to repeated mechanical deformation. The cyclic‐stretching record in Figure 5d was obtained at 100% strain over 50,000 s. The approximately 36 s period shown in the inset corresponds to approximately 1,400 stretch‐release cycles over this duration (n = 3).

### 4.9 Line Printing Resolution Characterization and Surface Morphology

The morphological features and printing resolution of the PmLMP lines were analyzed using a laser confocal microscope (VK‐X3000; Keyence, Osaka, Japan). To evaluate the printing quality according to various resolution settings, the 3D surface profiles, line widths, and cross‐sectional heights of the printed patterns were precisely measured. This analysis allowed for the quantitative assessment of the ink's fidelity and the consistency of the printing process.

### 4.10 Batch‐Compatible Production of Wound Healing Patch Application

To demonstrate the feasibility of scalable manufacturing, the wound healing patches were fabricated using an automated pick‐and‐place (PnP) machine (Neo Den YY1; Zhejiang Neo Den Technology Co., Ltd., China). The circuit was designed to incorporate an ATtiny85 microcontroller (MCU), four 640 nm red LEDs (0402 size), and a 150 Ω resistor (0603 size). These components were precisely mounted onto a VHB (Very High Bond) substrate, which served as a flexible base. Following the PnP assembly, the entire device was encapsulated with Ecoflex to provide a robust, waterproof, and biocompatible protective layer, ensuring stable operation during skin‐attachable applications.

### 4.11 In Vivo Wound Healing Evaluation

For in vivo wound healing evaluation, the therapeutic efficacy of the wearable LED patch was investigated using a murine full‐thickness excisional wound model. The animal study protocol was approved by the Institutional Animal Care and Use Committee (IACUC) at Yonsei University (approval number: IACUC‐A‐202605‐2260‐01), ensuring compliance with the guidelines for the humane care and use of laboratory animals. After depilation and sterilization of the dorsal skin with iodine solution, circular full‐thickness skin wounds with a diameter of 0.5 cm were created on the dorsum of mice using a biopsy punch. Mice were randomly assigned to either the control group or the LED treatment group. For the LED treatment group, the flexible LED patch was conformally attached to the wound site immediately after wound creation and secured to maintain stable contact with the skin. The LED patch was applied only transiently during each scheduled irradiation session and was removed immediately after irradiation. Following each treatment session, both the control and LED‐treated wounds were covered with sterile gauze under identical conditions. The wounds were irradiated according to the predetermined illumination protocol, while control wounds received no light treatment. Digital photographs of the wound areas were acquired at days 0, 3, 5, and 7 post‐wounding. Wound closure was quantified by measuring the residual wound area using image analysis software and expressed as a percentage of the initial wound area. At day 7 post‐wounding, mice were euthanized, and wound tissues together with the surrounding skin were harvested for histological and immunohistochemical analyses. Excised tissues were fixed in 4% paraformaldehyde, embedded in paraffin, and sectioned for staining. Hematoxylin and eosin (H&E) staining was performed to assess overall tissue morphology and re‐epithelialization. Masson's trichrome (MT) staining was used to evaluate collagen deposition and extracellular matrix remodeling. Immunohistochemical staining for PECAM‐1 (CD31) (Thermo Fisher Scientific, Cat. No. 14‐0311‐82) was conducted to assess neovascularization, while F4/80 (Thermo Fisher Scientific, Cat. No. 14‐4801‐82) staining was performed to evaluate macrophage infiltration within the wound tissue. Quantitative analyses of collagen density, micro vessel formation, and macrophage accumulation were performed using image analysis software based on stained tissue sections. All quantitative data were obtained from at least four independent animals per group. The entire experiment was repeated independently to ensure reproducibility and reliability of the results.

### 4.12 ECG, EMG Electrode Fabrication

The electrode geometries were designed using AutoCAD and fabricated by cutting OHP films with a laser cutting system (VLS 2.30; Universal Laser Systems) to create shadow masks. Using these masks, the PmLMP ink was printed onto textile and Tegaderm substrates, respectively. To enhance the signal acquisition, LiCl‐based hydrogel was integrated into the sensing electrode areas to ensure low interfacial impedance and stable skin contact. The remaining exposed interconnects were encapsulated with Ecoflex to provide insulation and protection against mechanical deformation and moisture. To synthesize the polyacrylamide‐LiCl (PAAm‐LiCl) hydrogel, a precursor solution was obtained by dissolving acrylamide (AAm) monomer (146.35 mg mL^−^^1^), N,N′‐methylenebis(acrylamide) (MBAA; 0.07% of AAm), ammonium persulfate (APS; 0.1% of AAm), 0.4 µL mL^−^^1^ of N,N,N′,N′‐tetramethylethylenediamine (TEMED), and LiCl in DI water. The solution was poured into the mold with a thickness of 3 mm. Then, the PAAm‐LiCl hydrogel was cured for 4 h at room temperature [82].

### 4.13 ECG, EMG Sensing & Analysis

All human EMG and ECG measurements were approved by the Institutional Review Board of Yonsei University (approval no. 7001988‐202607‐HR‐3279‐02) and were conducted after written informed consent was obtained from all participants.

For the acquisition and processing of electrophysiological signals, an integrated wireless system was developed using the BioAmp EXG Pill (OpenBCI) and an ESP32 microcontroller. The BioAmp EXG Pill served as the analog front‐end to amplify and filter the raw biopotentials, which were subsequently digitized by the ESP32's analog‐to‐digital converter (ADC). To ensure low‐power consumption and seamless data transmission, a Bluetooth Low Energy (BLE) protocol was implemented for wireless communication with external interfaces. The practical utility of the sensing system was demonstrated through two distinct applications. First, a human‐machine interface (HMI) was established where EMG signals were processed to control the movement and direction of an RC car via BLE commands. Second, a custom real‐time ECG analysis software was developed using Python within the VS Code environment. This application featured a graphical user interface (GUI) for the real‐time visualization of ECG waveforms and integrated a peak‐detection algorithm to calculate heart rate (BPM) and analyze cardiac intervals. This comprehensive setup successfully demonstrated the system's capability for both interactive control and clinical healthcare monitoring.

### 4.14 Gravimetric Evaporation Analysis

Evaporation of DI water and DI water/ethanol droplets was tracked by drop‐casting a fixed volume onto a glass substrate placed on a precision analytical balance (XSR205DUV, Mettler Toledo). The droplet mass was recorded at fixed time intervals under ambient conditions, and the resulting mass–time curves were used to compare the evaporation behavior of the two solvent systems.

### 4.15 µ‐PIV Post‐Processing and Radial‐Flow Decomposition

PIVlab vector fields were converted to physical units using the measurement calibration and frame interval used in the main acquisition. The velocity fields were drift‐corrected by subtracting the spatial mean velocity before radial analysis. The droplet center was determined from the symmetry of the time‐averaged velocity field. Each velocity vector was decomposed into radial and azimuthal components about this center, with positive radial velocity defined as outward, toward the contact line, and negative radial velocity defined as inward. The radial velocity component was then azimuthally averaged in equal‐width radial bins to obtain the radial velocity profile as a function of radial position. The same decomposition was applied to early, mid, and late temporal windows during drying to evaluate the time evolution of the internal flow.

### 4.16 DFT (Density Functional Theory) and NCI Analysis of PVA–Ga2O3 Hydrogen Bonding

To evaluate whether PVA hydroxyl groups can interact with hydroxylated gallium oxide surface sites through hydrogen bonding, we performed density functional theory calculations using isopropanol as a molecular model of the PVA repeat unit and Ga(OH)_3_ as a hydroxylated Ga_2_O_3_ surface‐site model. B3LYP‐D3(BJ) was used to account for dispersion‐corrected non‐covalent interactions. Geometry optimization was performed using the def2‐SVP basis set, followed by single‐point energy calculations using def2‐TZVP. The isolated hydrogen‐bonded complex was further re‐optimized at the def2‐TZVP level. Basis‐set superposition error was corrected using the Boys–Bernardi counterpoise method, and MP2/def2‐TZVP calculations were performed as an additional cross‐check. The optimized complex exhibited a near‐linear O─H···O hydrogen bond between the PVA hydroxyl model and a surface oxygen of the hydroxylated gallium oxide model. The O···O distance was 3.13 Å, and the O─H···O angle was 169°. The counterpoise‐corrected interaction energy was −23.2 kJ mol^−^^1^ at the B3LYP‐D3(BJ)/def2‐TZVP level and −18.5 kJ mol^−^^1^ at the MP2/def2‐TZVP level, indicating a moderate hydrogen‐bonding interaction. Non‐covalent interaction analysis further showed an attractive interaction feature along the O─H···O axis, and the RDG vs. sign(λ_2_)ρ plot exhibited an attractive feature at sign(λ_2_)ρ ≈ −0.014 a.u. These calculations are intended to support the feasibility of PVA–Ga_2_O_3_ hydrogen bonding at hydroxylated gallium oxide surface sites. Because the model uses a small hydroxylated cluster rather than a periodic amorphous oxide surface, the calculated interaction energies should be interpreted as molecular‐level supporting evidence rather than as quantitative adhesion energies for the full PVA/liquid‐metal oxide interface.

### 4.17 Statistical Analysis

GraphPad Prism (GraphPad Software Inc., USA) and Origin (OriginLab Corporation) were used for data analysis and plotting. Data presentation, sample sizes (n), and the meaning of error bars are specified for the relevant panels in the figure captions. Prior to parametric analysis, data distributions were evaluated for normality using the Shapiro–Wilk test. Zeta‐potential and adhesion‐stress comparisons among formulations (Figure 2g,h) were analyzed using one‐way analysis of variance (ANOVA) followed by Tukey's multiple‐comparisons test. Wet masses before and after supernatant removal were compared using a paired two‐tailed Student's t‐test for three matched ink batches (Figure S1e). The solvent‐composition and substrate‐temperature experiment (Figure S13b) was analyzed using two‐way ANOVA, with solvent ratio and temperature as factors, followed by Tukey's multiple‐comparisons test comparing solvent ratios within each temperature. The conductivity comparison between the single‐ and binary‐solvent formulations (Figure 4j) and the endpoint‐wise comparisons between the control and LED‐treated groups (Figure S22b) were analyzed using one‐way ANOVA. Wound closure over time (Figure 6d) was analyzed using two‐way ANOVA with treatment group and time as factors, followed by Tukey's multiple‐comparisons test. Panels without a reported inferential comparison are interpreted descriptively. Statistical significance is denoted by *p < 0.05, **p < 0.01, ***p < 0.001, and ****p < 0.0001; N.S. denotes not significant.

## Author Contributions

J. Seo and T.Y. Kim conceived and supervised the study. K.‐Y. Yook designed and performed most of the experiments and data analysis. S. Kim conducted ink concentration optimization experiments. C. Kim, M. Kim, M. Kang, H. Jang, and K. Son conducted the ECG and EMG experiments. Y. Lee and K. Park drew the schematic illustration. S.Y. Han and S.‐W. Cho conducted rheology analysis of PmLMP. G.‐H. Lee contributed to the experimental design and interpretation of data associated with Marangoni and capillary effects. J. Seo, T.Y. Kim, and K.‐Y. Yook wrote the manuscript with input from all authors.

## Conflicts of Interest

The authors declare no conflicts of interest.

## Supporting information




**Supporting File 1**: advs78062‐sup‐0001‐SuppMat.docx.

**Supporting File 2**: advs78062‐sup‐0002‐VideoS1.mp4.

**Supporting File 3**: advs78062‐sup‐0003‐VideoS2.mp4.

**Supporting File 4**: advs78062‐sup‐0004‐VideoS3.mp4.

**Supporting File 5**: advs78062‐sup‐0005‐VideoS4.mp4.

**Supporting File 6**: advs78062‐sup‐0006‐VideoS5.mp4.

**Supporting File 7**: advs78062‐sup‐0007‐VideoS6.mp4.

## Data Availability

The data that support the findings of this study are available from the corresponding author upon reasonable request.
