# Supplementary material for: Synergistic Mechano‐Fluidic Self‐Fusion of Poly(Vinyl Alcohol)‐Modified Liquid‐Metal Particles for Post‐Treatment‐Free Soft Electronics
Source: Adv Sci (Weinh). 2026 Oct 6:e78062. Online ahead of print. doi: 10.1002/advs.78062 (PMC13643113; doi:10.1002/advs.78062)
Supplement: Supplementary file 1 [file ADVS-9999-e78062-s003.docx]

**Synergistic Mechano-Fluidic Self-Fusion of Poly(vinyl alcohol)-Modified Liquid-Metal Particles for Post-Treatment-Free Soft Electronics**

*Keun-Young Yook^1^, Sangwon Kim^1^, Chansoo Kim^1^, Minkyong Kang^1^, Yurim Lee^1^, Mido Kim^1^, Kijun Park^2^, Seung Yeop Han^3,4^, Seung-Woo Cho^3,5,6^, Gun-Hee Lee*^7^*, Kayoung Son^1^, Hanbit Jang^1^, Tae Young Kim^1*^, Jungmok Seo^1*^*

^1^School of Electrical and Electronic Engineering, Yonsei University, 50 Yonsei-ro, Seodaemun-gu, Seoul 03722, Republic of Korea

^2^Department of Medicine, Brigham and Women’s Hospital Harvard Medical School, MA 02139, USA

^3^Department of Biotechnology, Yonsei University, Seoul 03722, Republic of Korea

^4^Department of Biomaterials Science and Engineering, Yonsei University, Seoul 03722, Republic of Korea

^5^CellArtgen Inc., Seoul 03722, Republic of Korea

^6^Center for Nanomedicine, Institute for Basic Science (IBS), Seoul 03722, Republic of Korea

^7^Department of Biomedical Engineering, Ulsan National Institute of Science and Technology (UNIST), UNIST-gil 50, Ulju-gun, Ulsan, Republic of Korea

E-mail: jungmok.seo@yonsei.ac.kr (Prof. J. Seo), taeyoung.kim92@yonsei.ac.kr (Dr. T.Y. Kim)

Keywords: Polymer-shelled LMP, Marangoni-driven Self-fusion, Post-treatment-free, Instant conductivity, Enhanced wettability

**Figure S1. PmLMP inks and centrifugation-associated mass change. a** Final DI_LMP, PVA_LMP, and PmLMP dispersions. **b** Cross-sectional scanning electron microscopy (SEM) images of LM, LMP, PVA_LMP, and PmLMP (scale bar, 10 µm). **c** Top-view SEM images before and after centrifugation (scale bars, 100 µm). **d** Representative balance readings before centrifugation and after supernatant removal. **e** Corresponding wet masses before centrifugation and after supernatant removal. Bars show the mean, and error bars indicate SD for three independently prepared ink batches measured before and after supernatant removal (n = 3 matched batches); paired two-t*ailed Student’s t-test, p = 1.49 × 10⁻⁵ (****p < 0.0001).*

**Figure S2. Structural and compositional characterization of LMP after sonication in DI water. a** Transmission electron microscopy (TEM) images of LMP sonicated in DI water (scale bar: 20 nm). **b** Energy-dispersive X-ray spectroscopy (EDS) data of LMP sonicated in DI water.

**Figure S3. Interfacial characterization of the PVA-modified liquid metal particles (PmLMPs).** **a** Representative TEM image of a PmLMP showing the overall particle morphology (scale bar = 25 nm). **b** Magnified TEM image of the particle interface, in which the outer PVA-derived organic layer and the underlying native oxide layer are annotated. The PVA-rich organic layer is observed as an ultrathin outer shell, while the native oxide layer is located directly beneath it at the liquid-metal surface (scale bar = 20 nm). **c** Scanning transmission electron microscopy (STEM) image. **d** STEM-EDS elemental maps of C, O, Ga, and In obtained from the region shown in **c**, confirming the peripheral distribution of the PVA-related carbon signal and the oxide-associated oxygen signal around the liquid-metal particle. **e** EDS line-profile analysis acquired across the particle interface. The carbon-rich outer region corresponds to the PVA-derived organic layer, whereas the subsequent increase in O and Ga signals indicates the native Ga₂O₃ oxide shell beneath the PVA layer.

**Figure S4. DFT and NCI analyses supporting hydrogen bonding between PVA hydroxyl groups and hydroxylated Ga₂O₃ surface sites. a** Optimized hydrogen-bonded complex between the PVA hydroxyl model compound, isopropanol, and a hydroxylated gallium oxide surface-site model, Ga(OH)₃. The PVA-model O–H group donates a hydrogen bond to a surface oxygen of the hydroxylated Ga₂O₃ model, giving an O···O distance of 3.13 Å and an O–H···O angle of 169°. The counterpoise-corrected interaction energy is −23.2 kJ mol⁻¹ at the B3LYP-D3(BJ)/def2-TZVP level and −18.5 kJ mol⁻¹ at the MP2/def2-TZVP level. **b** Non-covalent interaction (NCI) analysis of the hydrogen-bonded complex. The NCI isosurface shows an attractive interaction feature along the O–H···O axis, and the RDG versus sign(λ₂)ρ plot exhibits an attractive feature at sign(λ₂)ρ ≈ −0.014 a.u., consistent with a moderate O–H···O hydrogen-bonding interaction. [1-4]

**Figure S5. Wettability characteristics and surface tension analysis. a** Optical images of contact angle measurements for the control group, DI_LMP, on various elastomeric substrates, showing higher contact angles than PmLMP on the same substrates (n = 2). **b** Optical images of PmLMP on the same substrates, showing significantly reduced contact angles due to the thin PVA coating. **c** Zisman-type plot of cos $\theta$versus substrate surface energy ($\gamma_{SV}$) fitted with the equation of state, demonstrating a marked reduction in calculated liquid surface tension ($\gamma_{LV}$) from ~72.8 mN m⁻¹ (DI_LMP) to ~54.6 mN m⁻¹ (PmLMP) for the effective lowering of the thermodynamic energy barrier by the PVA shell.

**Figure S6. Comparison of electromechanical behavior under stretching.** (i) SEM image of the stretched DI_LMP region; (ii) Ga EDS mapping of the crack region; (iii) SEM image of the stretched PVA_LMP region; (iv) Ga EDS mapping of the crack region; (v) SEM image of the stretched PmLMP region; (vi) corresponding EDS mapping confirming the presence of Ga even in the stretched region, indicating preserved electrical conductivity. (Scale bars: 5 µm)

**Figure S7. Cross-sectional SEM analysis of PVA adhesion and polymer bridging structure. a,b** Cross-sectional SEM images showing adhesion of PVA to the substrate (scale bar: 5 µm). **c, d** Cross-sectional SEM images showing the preservation of the polymer bridging structure in PVA (scale bar: 5 µm).

**Figure S8. Cross-sectional SEM analysis of pristine DI_LMP control films. a** Cross-sectional SEM image of a spray-printed DI_LMP film showing loosely stacked liquid-metal particles with limited adhesion to the substrate (scale bar = 5 µm). **b,c** Cross-sectional SEM images of drop-cast DI_LMP samples showing large aggregated liquid-metal domains formed during or after particle preparation. The aggregated particles show weak interfacial attachment to the substrate, consistent with the low adhesion and surface-tension-driven coalescence behavior of pristine LMPs (scale bars = 100 µm).

**Figure S9. Cross-sectional SEM and EDS analysis of PVA_LMP control films.** **a** Cross-sectional SEM image of a spray-coated PVA_LMP film showing a PVA-rich, entangled particle network. (Scale bar = 20 µm) **b** Corresponding EDS elemental map of **a** showing carbon-rich regions associated with the PVA overlayer and polymer bridging. **c** Cross-sectional SEM image of a drop-cast PVA_LMP film showing web-like polymer-rich structures between liquid-metal particles. (Scale bar = 5 µm) **d** Corresponding EDS elemental map of **c** confirming that the web-like fibrillar regions are enriched in carbon and oxygen, consistent with accumulated PVA chains around the liquid-metal particles.

**Figure S10. Morphological differences between low- and high–air-to-liquid ratio (ALR) spray printing of PmLMP. a** Spray printing of PmLMP on Tegaderm at low ALR, showing excessive solvent content that results in a visibly wet substrate. **b** Spray printing of PmLMP on Tegaderm under optimized ALR conditions, where an appropriate amount of solvent rapidly evaporates, enabling immediate sintering. **c** Post-printing comparison of the two cases, revealing that low-ALR printing leads to residual solvent retention and consequently disordered and highly non-uniform deposition. **d** Direct manifestation of electrical conductivity achieved by spray printing PmLMP on a latex glove at high ALR.

**Figure S11. Effect of sonication amplitude on PmLMP printing characteristics.** a SEM top-view images of printed patterns prepared under different sonication amplitudes. **b** Variation of line resistance as a function of sonication amplitude for patterns printed over an area of 2 × 1 cm² (n = 4). *Bars show mean resistance, and error bars indicate SD.* No infere*ntial statistical comparison is reported. No measurable conduction was observed at 100% sonication amplitude.*

**Figure S12. Sonication-amplitude-dependent morphological and compositional evolution of PmLMP. a** Top-view SEM image of the sample prepared at 50% sonication amplitude, showing irregular surface deposits and aggregated features on the liquid-metal particles (scale bar = 10 µm). **b** SEM image of the same sample after dilution in IPA, revealing nanoscale particle/deposit structures associated with high-amplitude sonication (scale bar = 500 nm). **c** Electron microscopy image and corresponding EDS elemental maps of the sample prepared at 100% sonication amplitude, showing Ga- and O-rich features with negligible In signal. These observations are consistent with the formation of oxide- or hydroxide-rich byproducts under high-amplitude sonication, which can interfere with liquid-metal particle contact and coalescence.

**Figure S13. Substrate-temperature and solvent-composition effects on the conductivity of spray-printed PmLMP films. a** Photograph of PmLMP spray printing onto a substrate placed on a temperature-controlled hot plate. **b** Electrical conductivity of films printed at room temperature (RT), 50 °C, and 100 °C using DI water/ethanol volume ratios of 1:1, 2:1, 3:1, and 4:1. Increasing the substrate temperature produced a modest conductivity increase, consistent with accelerated solvent evaporation. The 3:1 ratio at RT (blue) was selected as the general optimized condition because it provided high conductivity while remaining compatible with temperature-sensitive substrates. Data are presented as mean ± SD (n = 4 per condition, except n = 5 for the 1:1 DI water/ethanol ratio at 50 °C). Error bars represent SD. Statistical significance was assessed using two-way ANOVA with solvent ratio and substrate temperature as factors, followed by Tukey’s multiple-comparisons test comparing solvent ratios within each temperature. *Only significant comparisons are shown. Asterisks indicate adjusted P values: *p < 0.05, **p < 0.01, ***p < 0.001, and ****p < 0.0001.*

**Figure S14. Surface morphology evolution of PVA_LMP and PmLMP under repeated printing cycles.** **a** Top-view images of PVA_LMP showing particle accumulation without fracture (scale bar: 50 µm). **b** Top-view images of PmLMP showing particle fracture and percolation network formation (scale bar: 50 µm).

**Figure S15. Evaporation behavior and internal Marangoni flow of DI water and DI water/ethanol binary solvent droplets.** **a** Time-dependent weight change of DI water and DI water/ethanol droplets measured using a precision balance; the binary solvent shows faster mass loss, consistent with the higher volatility of ethanol. **b** Time-averaged in-plane velocity field of the DI water/ethanol droplet (µ-PIV); color encodes the radial velocity v_r (red, outward; blue, inward) and the cross marks the droplet center. **c** Azimuthally-averaged radial velocity *v*_r_ versus radial position *r* at early, mid, and late stages of drying. **d** Schematic of the inferred solutal Marangoni loop (outward surface flow, inward basal return); dashed line, imaging focal plane.

**Figure S16. Effect of Marangoni flow on particle packing and percolation network formation. a** Drop-cast DI solvent sample without Marangoni flow, showing loosely packed particles with low packing density (scale bar: 10 µm). **b** Drop-cast binary solvent sample exhibiting Marangoni flow, where densely packed particles form a percolation network (scale bar: 5 µm). Related evaporation-driven particle assembly has been reported in a distinct liquid-metal-particle film system.[5]

**Figure S17. Reproducibility and resistance of high-resolution PmLMP lines. a–d** Laser-confocal profiles (VK-X3000, Keyence) of nominal 50, 100, 300, and 500 µm lines. e Measured resistance of 1 cm-long printed lines at each nominal linewidth. The mean and SD were calculated at each lateral position across the five profiles. In e, bars represent the mean resistance and error bars indicate SD (n = 5 printed lines per nominal linewidth). *The data are presented descriptively; no inferential statistical comparisons are reported.*

**Figure S18. Electrical, mechanical, and processing performance of PmLMP-based conductors. a** Comparison of electrical conductivity with previously reported conductive materials. **b** Summary of spray-coating conditions. **c** Electrical conductivity of printed patterns on different substrates (n = 3). *No inferential statistical comparison was performed*.

**Figure S19. Effect of oxygen-plasma treatment on PmLMP adhesion to nonpolar elastomers.** Photographs of PmLMP traces on polydimethylsiloxane (PDMS, left) and Ecoflex (right), with (top) or without (bottom) oxygen-plasma treatment, during manual stretching; insets show enlarged printed regions. Plasma-treated substrates retained more uniform, continuous coatings, whereas untreated substrates showed nonuniform deposition, consistent with poorer wetting and adhesion.

**Figure S20. PmLMP-based electronic-device fabrication and mechanical deformability. a** Chip-programming (bootloader) circuit implemented using PmLMP. **b** Large-area LED-control arrays fabricated using PmLMP. **c** Stretching of a fabricated device.

**Figure S21. Fabrication and deformability of the wound-healing light-emitting-diode (LED) patch. a** Device fabricated by printing the circuit on a VHB substrate, mounting the chip, and encapsulating it with Ecoflex. **b** Device during operation. **c** Device after flipping and folding in half.

**Figure S22. Histological and immunohistochemical evaluation of LED-patch-assisted wound healing. a** Representative histological and immunohistochemical images of wound tissues at day 7, including hematoxylin and eosin (H&E) staining (pink, cytoplasm; purple, nuclei), Masson’s trichrome (MT) staining (blue, collagen), PECAM-1 staining (brown, endothelial cells; blue, nuclei), and F4/80 staining (brown, macrophages; blue, nuclei). **b** Quantitative analysis of collagen density, neovascularization (PECAM-1-positive area), and macrophage infiltration (F4/80-positive area), normalized to the control group. Data in b are presented as mean ± SD (n = 4 animals per group); error bars indicate SD. *Each endpoint was compared between the control and LED-treated groups using one-way ANOVA. *p < 0.05, **p < 0.01.*

**Figure S23. Electromyography (EMG) and electrocardiography (ECG) performance of PmLMP + LiCl electrodes. a** Forearm EMG measurement setup using commercial 3M™ Ag/AgCl electrodes (control) and fabricated PmLMP + LiCl electrodes. **b** Representative EMG waveforms and corresponding signal-to-noise ratios of 15.62 and 36.0 dB for the commercial and PmLMP + LiCl electrodes, respectively. **c** EMG signals during grasping and a lower-intensity hand gesture, showing differentiated amplitudes. **d** Photograph of real-time ECG monitoring. **e,f** Representative ECG waveforms acquired using **e**, a commercial 3M™ Ag/AgCl electrode, and **f**, a PmLMP + LiCl electrode, showing distinguishable P-Q-R-S-T features.

**Figure S24. Evaluation of *in vivo* electrophysiological sensing performance of the large-area printed PmLMP electrode. a** Schematic workflow of the EMG-based wireless human-machine interface (HMI) system. EMG signals acquired from the forearm are filtered in the 74.5–149.5 Hz band and transmitted through a Bluetooth Low Energy (BLE) module to generate control commands for a remote-controlled (RC) car. **b** Demonstration of gesture-dependent RC-car control. Distinct hand gestures are mapped to Stop, Go, Turn Left, and Turn Right commands. **c** Schematic workflow of the ECG monitoring system. ECG signals acquired from the chest are filtered in the 0.5–44.5 Hz band and wirelessly transmitted through a BLE module for heart-rate monitoring.

**Supplementary Note S1**

**Supplementary Note S1. Impact-energy consistency check for the AFM-measured rupture threshold.** The atomic force microscopy (AFM) force –distance curve (Figure 3e) gives the shell-rupture force F_yield_ = 266.08 nN and, over the indentation depth δ at rupture, a rupture energy E_c_ ≈ ½·F_yield_·δ ≈ 3.8 × 10⁻¹⁴ J. Assuming the reported 30 psi carrier-gas pressure is gauge pressure, the upstream-to-ambient absolute pressure ratio (≈ 3.0) exceeds the critical ratio for choked air flow (~1.89), so ideal choked-flow scaling suggests a carrier-gas velocity on the order of 10² m s⁻¹. Because the particles are entrained in a two-phase jet and travel more slowly than the gas, we adopt a deliberately conservative particle velocity of ~20 m s⁻¹. Here, D ≈ 1.18 µm is the measured mean diameter of the optimized DI water/ethanol (3:1 v/v) spray formulation (Figure 4i) and is used as the representative particle diameter in this consistency check; 309.7 nm is the DI-only control value, and R = 250 nm in Figure 4h is an illustrative capillary-model radius. For the representative particle (D ≈ 1.18 µm, m ≈ 5.4 pg), the impact kinetic energy is E_k_ = ½mv² ≈ 1.1 × 10⁻¹² J, exceeding E_c_ by approximately 25–30 times; equivalently, the critical impact velocity required to supply E_c_ is only v_c_ = (2E_c_/m)½ ≈ 4 m s⁻¹, well below the conservative spray-particle velocity estimate. A force-based estimate gives the same order-of-magnitude conclusion: if the impact energy is dissipated over a deformation length comparable to the AFM indentation depth, the work-equivalent average impact force is on the order of several µN, more than an order of magnitude larger than the 266.08 nN rupture force. Because E_k_ exceeds E_c_ by an order of magnitude, particle-to-particle variation in the single-particle indentation depth δ does not change this conclusion. This E_k_-versus-E_c_ criterion is shown schematically in Figure 3h and is consistent with the measured conductivity saturation above ~30 psi (Figure 3k) and its decrease at the tested 6 cm working distance (Figure 3l). Particles that do not fully rupture during impact may remain partially encapsulated and are further consolidated by the secondary Marangoni/capillary mechanism. Although the main text provides a stress-based plausibility estimate using an explicitly assumed 11 nm contact radius, we do not use that estimate as the primary impact criterion because the transient contact area was not measured.

**Supplementary references**

[1] Smith, D. G.; Burns, L. A.; Simmonett, A. C.; Parrish, R. M.; Schieber, M. C.; Galvelis, R.; Kraus, P.; Kruse, H.; Di Remigio, R.; Alenaizan, A. PSI4 1.4: Open-source software for high-throughput quantum chemistry. *The Journal of chemical physics* **2020**, *152* (18).

[2] Becke, A. D. Density‐functional thermochemistry. III. The role of exact exchange. *The Journal of chemical physics* **1993**, *98* (7), 5648-5652.

[3] Lee, C.; Yang, W.; Parr, R. G. Development of the Colle-Salvetti correlation-energy formula into a functional of the electron density. *Physical review B* **1988**, *37* (2), 785.

[4] Grimme, S.; Antony, J.; Ehrlich, S.; Krieg, H. A consistent and accurate ab initio parametrization of density functional dispersion correction (DFT-D) for the 94 elements H-Pu. *The Journal of chemical physics* **2010**, *132* (15).

[5] Boley, J. W.; Hyun, S.-H.; White, E. L.; Thompson, D. H.; Kramer, R. K. Hybrid self-assembly during evaporation enables drop-on-demand thin film devices. *ACS Applied Materials & Interfaces* **2016**, *8* (50), 34171-34178.
